# Development of Ni-Sr(V,Ti)O_3-δ_ Fuel Electrodes for Solid Oxide Fuel Cells

**DOI:** 10.3390/ma15010278

**Published:** 2021-12-30

**Authors:** Bernardo F. Serôdio Costa, Blanca I. Arias-Serrano, Aleksey A. Yaremchenko

**Affiliations:** 1 Department of Materials and Ceramic Engineering, CICECO—Aveiro Institute of Materials, University of Aveiro, 3810-193 Aveiro, Portugal; bernardo.costa.90@gmail.com; 2 Leibniz Institute for Plasma Science and Technology, Felix-Hausdorff-Str. 2, 17489 Greifswald, Germany

**Keywords:** solid oxide fuel cell, anode, titanate, vanadate, electrode polarization, electrical conductivity, thermal expansion

## Abstract

A series of strontium titanates-vanadates (STVN) with nominal cation composition Sr_1-*x*_Ti_1-*y*-*z*_V*_y_*Ni*_z_*O_3-δ_ (*x* = 0–0.04, *y* = 0.20–0.40 and *z* = 0.02–0.12) were prepared by a solid-state reaction route in 10% H_2_–N_2_ atmosphere and characterized under reducing conditions as potential fuel electrode materials for solid oxide fuel cells. Detailed phase evolution studies using XRD and SEM/EDS demonstrated that firing at temperatures as high as 1200 °C is required to eliminate undesirable secondary phases. Under such conditions, nickel tends to segregate as a metallic phase and is unlikely to incorporate into the perovskite lattice. Ceramic samples sintered at 1500 °C exhibited temperature-activated electrical conductivity that showed a weak p(O_2_) dependence and increased with vanadium content, reaching a maximum of ~17 S/cm at 1000 °C. STVN ceramics showed moderate thermal expansion coefficients (12.5–14.3 ppm/K at 25–1100 °C) compatible with that of yttria-stabilized zirconia (8YSZ). Porous STVN electrodes on 8YSZ solid electrolytes were fabricated at 1100 °C and studied using electrochemical impedance spectroscopy at 700–900 °C in an atmosphere of diluted humidified H_2_ under zero DC conditions. As-prepared STVN electrodes demonstrated comparatively poor electrochemical performance, which was attributed to insufficient intrinsic electrocatalytic activity and agglomeration of metallic nickel during the high-temperature synthetic procedure. Incorporation of an oxygen-ion-conducting Ce_0.9_Gd_0.1_O_2-δ_ phase (20–30 wt.%) and nano-sized Ni as electrocatalyst (≥1 wt.%) into the porous electrode structure via infiltration resulted in a substantial improvement in electrochemical activity and reduction of electrode polarization resistance by 6–8 times at 900 °C and ≥ one order of magnitude at 800 °C.

## 1. Introduction

Concerns about the disadvantages of traditional Ni–YSZ (YSZ = yttria-stabilized zirconia) anodes of solid oxide fuel cells (SOFCs) have boosted significant research activities on the development of alternative anode materials [1,2]. Ni–YSZ cermets have poor redox stability, and significant volume changes upon occasional nickel oxidation/re-reduction cycles cause irreversible microstructural degradation [3,4,5]. Nickel agglomeration is another common degradation mechanism that leads to a loss of electrical connectivity between Ni particles and a decrease in electrocatalytic activity [5,6]. In addition, Ni–YSZ anodes are readily poisoned by sulfur and suffer from carbon deposition, which are key issues for direct operation with hydrocarbon fuels [7,8].

Oxide ceramic materials such as SrVO_3_- and SrTiO_3_-based perovskites are considered as promising alternative components for anodes of hydrocarbon-fueled SOFC [2,8,9,10,11]. Perovskite-like strontium vanadate SrVO_3-δ_ exhibits a high metal-like electronic conductivity (1000 S/cm at 800 °C and p(O_2_) ~10^−20^ atm) [12,13,14,15,16] possibly combined with substantial oxygen-ionic conductivity implied by a non-negligible oxygen deficiency (δ = 0.08–0.10) at elevated temperatures [16,17]. (Sr,La)VO_3_-based anodes were demonstrated to possess excellent tolerance against sulfur poisoning (H_2_S concentration up to 5–10%) [18,19] and a high resistance to carbon deposition [19,20,21] in syngas- and methane-fed cells. Two main drawbacks hinder the potential of strontium vanadate-based phases to be used as electrodes in SOFCs. First, the high thermal expansion coefficient (22.7 ppm/K at 500–950 °C) limits the thermomechanical compatibility with common solid electrolytes [15,16,22]. The second drawback is its limited phase stability, which is restricted to reducing conditions with an upper p(O_2_) stability boundary corresponding to ~10^−15^ and 10^−17^ atm at 900 °C and 800 °C, respectively [12,15,22].

On the contrary, perovskite-type SrTiO_3_ is a semiconductor with a comparatively low electrical conductivity (0.02–0.09 S/cm at 800 °C and p(O_2_) ~10^−20^ atm) [23,24,25]. The electrical conductivity of SrTiO_3_ under reducing conditions can be improved by donor-type substitutions in one or both sublattices (e.g., by rare-earth cations into a Sr sublattice and by Nb^5+^ into a titanium sublattice) [10,25,26]. Moreover, SrTiO_3_-based ceramics exhibit a moderate thermal expansion coefficient (~11.7 ppm/K at 25–1100 °C [25]) and remarkable thermodynamic stability in a wide range of p(O_2_)-T conditions. Similar to the phases derived from SrVO_3_, SrTiO_3_-based anodes demonstrate a good tolerance to sulfur poisoning and a suitable coking resistance [10,27,28]. Still, a comparatively low electrical conductivity (<10 S/cm in the typical fuel electrode environment) [29,30,31] and poor intrinsic electrocatalytic activity towards fuel oxidation remain major disadvantages of strontium titanate-based components for SOFC anode applications [32,33].

A reasonable compromise between high electrical conductivity and phase stability can be reached by balancing the fractions of vanadium and titanium cations in the B sublattice [11,34]. In particular, SrV_1-*y*_Ti*_y_*O_3-δ_ solid solutions with moderate titanium content (0.3 ≤ *y* ≤ 0.5) showed electrical conductivity ≥ 20 S/cm at temperatures ≤ 900 °C, combined with a phase stability domain extended up to p(O_2_) of at least 10^−11^ atm at 900 °C [34]. In addition, increasing the titanium content was found to gradually suppress thermochemical expansion, thus improving the thermomechanical compatibility with solid electrolyte ceramics [34].

Although balanced electrical properties and phase stability are indispensable requirements of SOFC anodes, high electrocatalytic activity is equally essential to attain appropriate cell performance. As titanate-based electrodes exhibit insufficient activity towards anodic reactions, an increasing number of research works have focused on the enhancement of their electrochemical performance. Improved electrochemical activity was reported for Y-doped SrTiO_3_ anodes infiltrated with Ni [35,36,37,38], Ru combined with ceria [27], or nano-sized Pd catalyst [39], as well as for (Sr,La)TiO_3_ and Sr(Ti,Nb)O_3_ anodes infiltrated with Ni [40], Pd and ceria [41,42], or doped ceria and Cu [43]. More recently, the exsolution of catalytically active nanoparticles initially dissolved in the crystal lattice of a host oxide attracted great attention as an alternative strategy to enhance the activity of oxide-based electrodes. Available examples of SrTiO_3_-based electrodes include exsolution of B-site transition metal cations such as Ni [44], Fe [45], and Ru and Fe [46]. A successful exsolution process seemingly depends not only on the inherent reducibility of dissolved B-site cations, but also on the defect chemistry that might be the main driving force.

The present work explores the possibility of the formation of Ni-substituted Sr(Ti,V)O_3_-based solid solutions and their electrochemical performance as potential fuel electrode materials for SOFCs. The initial objective was to determine the specific role of A-site deficiency and B-site cation balance on the solubility of nickel in the host Sr(Ti,V)O_3_ lattice and its exsolution under operation conditions. To this end, the general cation composition was formulated as Sr_1-*α*_Ti_1-*β*(1+γ)_V*_β_*Ni*_βγ_*O_3-δ_ (STVN) and particular compositions were selected employing Taguchi-type experimental planning [47]. Based on previous results [34], the vanadium content was kept at 20–40 at.%. The experimental procedure relied on the conventional solid-state reaction route in reducing atmospheres combined with mechanical activation. Contrary to initial expectations, the high stability of the strontium orthovanadate intermediate phase did not allow the implementation of the synthesis of Ni-containing solid solutions at reasonably low temperatures. Therefore, the work was focused on the assessment of the phase evolution under reducing conditions, studies of thermal expansion and electrical conductivity of STVN ceramics, and characterization of STVN-based electrodes using electrochemical impedance spectroscopy.

## 2. Materials and Methods

### 2.1. Synthesis and Ceramic Processing

The cation compositions of the STVN series were formulated as Sr_1-*α*_Ti_1-*β*(1+γ)_V*_β_*Ni*_βγ_*O_3_ and selected by the Taguchi method of experimental planning [47] using three variables (*v* = *α*, *β*, *γ*) with three levels (*n* = 1, 2, 3), as summarized in Table S1 (see Supplementary Materials). The nine selected compositions and their corresponding notations used hereafter are listed in Table 1. All materials were prepared by conventional solid-state reaction route using SrCO_3_ (Sigma-Aldrich, Hamburg, Germany, >99.9%), TiO_2_ (anatase, Sigma-Aldrich, >99.9%), V_2_O_3_ (Sigma-Aldrich, >99.9%), and NiO (Sigma-Aldrich, >99.9%) as starting chemicals. The mixtures of precursors were mechanically activated in dry conditions at 600 rpm for 4 h using a Retsch PM 100 planetary mill (Retsch GmbH, Haan, Germany), tetragonal zirconia grinding jar (250 mL), and milling media (Tosoh balls Ø 10 mm; ball-to-powder weight ratio of 10:1) in a regime of 5 min milling followed by a 5 min pause; the direction of rotation was changed after each pause.

Mechanically activated precursor powders were subjected to thermal treatments in a controlled atmosphere of 10 vol.% H_2_ in N_2_. In order to assess the effects of temperature and time of thermal treatment on the phase formation, powdered samples were calcined at different temperatures between 800 and 1200 °C for 4–10 h, with intermediate regrinding in a mortar with ethanol when appropriate. The final powders were prepared by calcination of precursor mixtures at 1200 °C for 30 h in total (see discussion below). These as-synthesized powders were ball-milled with ethanol for 4 h at 150 rpm, dried, and then used for the preparation of ceramic samples and electrodes. The disk-shaped ceramic samples were compacted by uniaxial pressing (Ø 18 mm, thickness ~2 mm, 40 MPa) followed by isostatic pressing (230 MPa) and sintered in flowing 10% H_2_–N_2_ at 1450 °C for 10 h.

Sintered ceramic pellets were polished using SiC grinding paper (Buehler, Leinfelden-Echterdingen, Germany). The density of the prepared ceramics (ρ_exp_) was calculated from the geometric dimensions and masses of the polished samples. Rectangular bars (approximate dimensions: 1.5 × 2.5 × 12 mm) for electrical and dilatometric measurements were cut out of disk-shaped samples using a Struers Minitom precision cutting machine (Struers, Copenhagen, Denmark) with a diamond cut-off wheel. Powdered samples for X-ray diffraction (XRD) were prepared by grinding sintered ceramics in a mortar.

### 2.2. Characterization of Materials

XRD patterns of powdered samples were recorded using a PANalytical X’pert PRO diffractometer (CuK_α_ radiation, 2Θ range of 10–80°, PANalytical, Almelo, The Netherlands). Phase identification and quantification were carried out using X’Pert Highscore Plus software (PANalytical). The unit cell parameters were calculated using FullProf software (v.7.20, ILL, Grenoble, France) employing the profile matching method. Microstructural characterization of STVN ceramics was performed through scanning electron microscopy (SEM) using a Hitachi SU-70 microscope (Hitachi, Tokyo, Japan) equipped with a Bruker Quantax 400 detector (Bruker, Berlin, Germany) for energy dispersive spectroscopy (EDS) analysis. Thermal expansion of STVN ceramics between 25 and 1100 °C was studied using controlled-atmosphere dilatometry in a vertical Linseis L75 dilatometer on heating/cooling (3 °C/min) in a flowing 10% H_2_–N_2_ mixture.

The electrical conductivity (σ) of the STVN ceramics was measured as a function of temperature and oxygen partial pressure p(O_2_) using the four-probe DC technique with bar-shaped samples. Pt wires were used as probes and current collectors. Additionally, the end-face surfaces of the bars were covered with Pt paint (Heraeus CL11-5349, Hanau, Germany) to improve the electrical contact. DC was supplied by a Yokogawa 7651 source and the voltage between the potential probes was measured using an Agilent 34460A multimeter. The experiments were performed in a cooling regime in a flowing 10% H_2_–N_2_ atmosphere with stepwise decreases in temperature from 1000 to 300 °C and isothermally with a stepwise increase in p(O_2_) from 10^−20^ to 10^−16^ atm. The p(O_2_) was controlled by the composition of 10% (H_2_ + H_2_O)-N_2_ gas mixtures and measured using an electrochemical 8 mol.% yttria-stabilized zirconia oxygen sensor. The minimum equilibration time before each measurement was 30 min.

### 2.3. Fabrication and Electrochemical Characterization of Electrodes

The electrochemical activity of the STVN electrodes was evaluated using an electrolyte-supported symmetrical cell configuration. For the fabrication of solid electrolyte membranes, commercial yttria-stabilized zirconia (ZrO_2_)_0.92_(Y_2_O_3_)_0.08_ (8YSZ, Tosoh, Tokyo, Japan) powders were compacted uniaxially into disks (Ø 15 mm), sintered in air at 1600 °C for 10 h, and then polished down to a thickness of 1.3 mm. STVN inks were prepared using as-synthesized powders (30 vol.%) mixed with an organic vehicle composed of α-terpineol (solvent) and ethyl cellulose (binder) with additions of stearic acid as a dispersant agent (extra 3 wt.%). The ink components were blended in a planetary ball mill (Retsch S1 mill, nylon vial, tetragonal zirconia balls) for 4 h at 150 rpm. Circular electrodes with an effective area of 0.2 cm^2^ were brush-painted symmetrically on each side of the 8YSZ solid electrolyte disk and sintered in a flowing 10% H_2_–N_2_ gas mixture at 1100 °C for 2 h (heating/cooling rate of 3 °C/min) to consolidate the porous electrode layers with ~40–50 μm thickness.

The performance of the symmetrical half-cells was evaluated by electrochemical impedance spectroscopy (EIS) using an AUTOLAB PGSTAT 302 instrument (Eco Chemie, Utrecht, Netherlands) equipped with a frequency response analyzer (FRA2) module. Pt gauzes with Pt wires were used as current collectors. The experiments were performed in a humidified fuel atmosphere (~3% H_2_O–10% H_2_–N_2_) under zero-DC conditions. The EIS spectra were registered in the frequency range from 0.01 Hz to 1 MHz with an AC amplitude of 50 mV. After heating, the cell was held at 900 °C for ≥20 h before starting the experiment. Following that, the measurements were carried out in a cooling regime from 900 to 700 °C with a 50 °C step. The stabilization time at each step was ~1 h. A post-mortem SEM/EDS inspection was performed in order to examine the microstructure of the STVN electrodes after the EIS measurements.

### 2.4. Electrode Modifications by Infiltration

The modification of STVN electrodes with gadolinia-doped ceria Ce_0.9_Gd_0.1_O_2-δ_ (CGO) and metallic Ni was conducted via the infiltration procedure. Several aqueous solutions of metal nitrates were prepared using Ce(NO_3_)_3_·6H_2_O (Sigma-Aldrich, ≥99.9%), Gd(NO_3_)_3_·6H_2_O (Alfa Aesar, ≥99.9%), and Ni(NO_3_)_2_·6H_2_O (Sigma-Aldrich, ≥99.9%). The solutions contained metal cations in different proportions: (i) Ce:Gd = 9:1, (ii) Ce:Gd:Ni = 9:1:1, and (iii) Ce:Gd:Ni = 9:1:5. Careful infiltration of a solution into the porous electrode structure was followed by drying and thermal treatment at 900 °C in a 10% H_2_–N_2_ atmosphere for 2 h for decomposition of nitrates and formation of dispersed CGO and Ni particles. The load of infiltrated components was calculated from the change in the weight of the cells. When necessary, the infiltration process was repeated to achieve the target load in each electrode (~20–30 wt.%).

## 3. Results and Discussion

### 3.1. Synthesis and Phase Evolution

#### 3.1.1. Effect of Mechanochemical Treatment

The mechanical activation of the precursor mixtures resulted in a decrease in the intensity and a noticeable broadening of the main XRD reflections (Figure S1). This could be caused by a number of factors, including the reduction in the particle size, the lattice strain induced by high-energy milling, and even partial amorphization of initial crystalline phases. The onset of the target SrTiO_3_-based perovskite phase was observed for some compositions (Figure S1). Overall, the mechanochemical treatment resulted in a rather limited output in terms of the formation of the desired product but provided very good precursor homogenization, which was favorable for further synthetic process.

#### 3.1.2. Effect of Calcination Temperature

**Figure 1:**
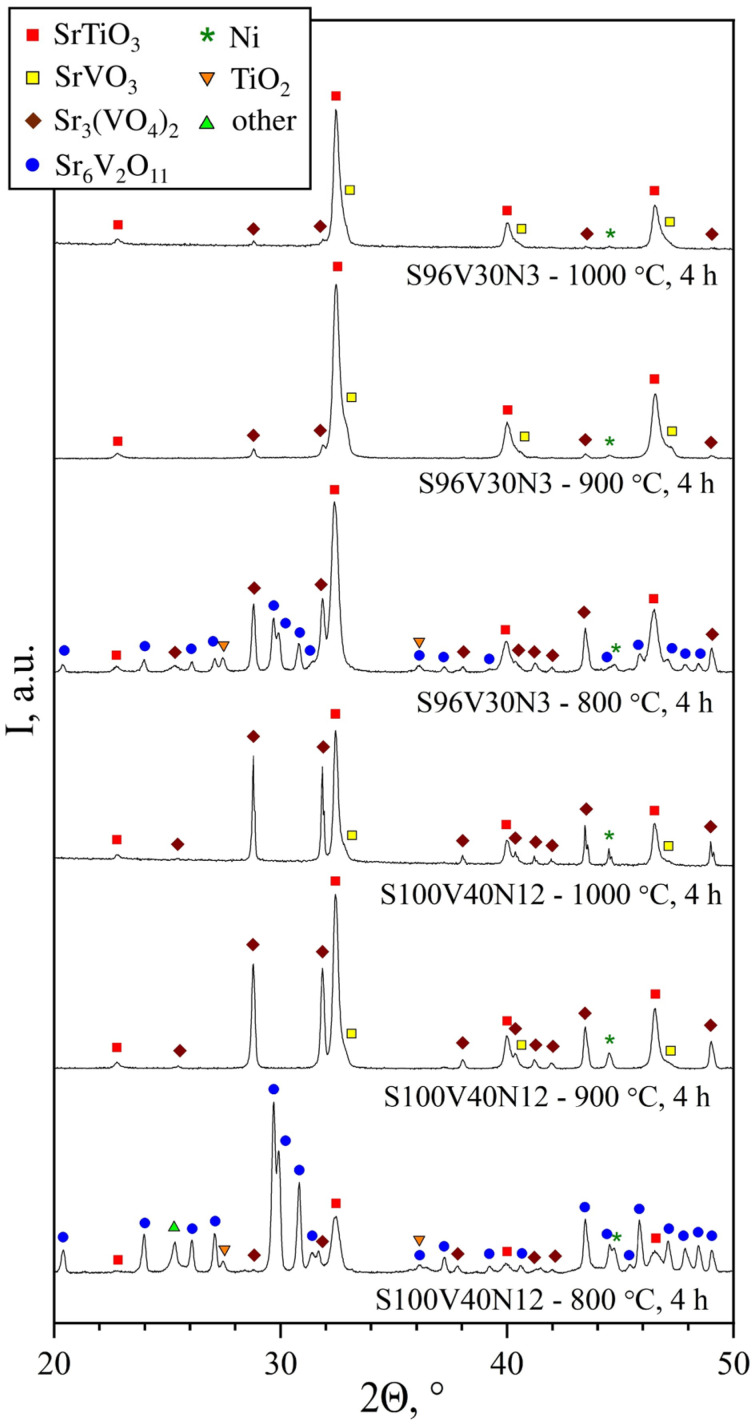
XRD patterns of S100V40N12 and S96V30N3 samples after calcination for 4 h in a flowing 10% H_2_–N_2_ atmosphere at 800, 900, and 1000 °C.

The mechanically-activated precursor mixtures were calcined for 4 h in a flowing 10% H_2_–N_2_ mixture at three different temperatures (800, 900, and 1000 °C) with the aim to find optimal conditions for the easy formation of the target perovskite phase. Figure 1 shows the XRD pattern of calcined samples of two selected compositions: S100V40N12 without A-site vacancies and with the highest nominal nickel content and S96V30N3 with A-site vacancies and low nickel content. The thermal treatment at 800 °C was not very effective; the corresponding XRD patterns revealed the presence of substantial amounts of secondary products, including Sr_3_(VO_4_)_2_, metallic Ni, traces of unreacted TiO_2_, and other phases. Increasing the temperature of calcination up to 900 °C substantially promoted the formation of the SrTiO_3_-based perovskite that became the main phase after this treatment. Nonetheless, all compositions still comprised substantial amounts of secondary phases including Sr_3_(VO_4_)_2_ (particularly in A-site stoichiometric compositions), the second perovskite phase based on SrVO_3_, and metallic Ni. The calcination at 1000 °C yielded very similar results, with only a minor decrease in the fraction of Sr_3_(VO_4_)_2_ impurity. The XRD patterns and estimated fractions of different phases for all STVN compositions after calcinations at 800–1000 °C can be found in Figures S2–S4. Analysis of the results revealed the correlations between the nominal composition and the fractions of secondary phases. The amount of segregated metallic nickel phase is rationally interrelated with the nominal nickel content. The fraction of Sr_3_(VO_4_)_2_ impurity increases with the increasing nominal Ni content but decreases with the increasing nominal A-site deficiency. Increasing vanadium content promotes the segregation of SrVO_3_-based perovskite, particularly for nominally Sr-deficient compositions. Note that, due to the very close lattice parameters of SrTiO_3_ and SrVO_3_ phases and, consequently, the overlapping of the XRD reflections, the detection and quantification of SrVO_3_ secondary phase was not always possible. In summary, the results of phase analysis imply that nickel does not tend to incorporate into the B-sublattice of Sr(Ti,V)O_3_ perovskites during the thermal treatment of mechanically-activated precursors in reducing atmospheres at 800–1000 °C but preferentially segregates in metallic form. This results in an excess of strontium, particularly in the compositions with a higher nominal nickel content, which, in turn, promotes the formation of a strontium orthovanadate Sr_3_(VO_4_)_2_ phase.

#### 3.1.3. Phase Evolution in Consecutive Calcination Steps

As direct calcinations were not efficient for the preparation of phase-pure perovskite-type solid solutions, the evolution of the phase compositions was assessed in consecutive thermal treatment steps of pelletized samples in flowing 10% H_2_–N_2_ at 800–1100 °C. After each calcination, the samples were ground in a mortar with ethanol, compacted again, and subjected to the next step of thermal treatment.

**Figure 2:**
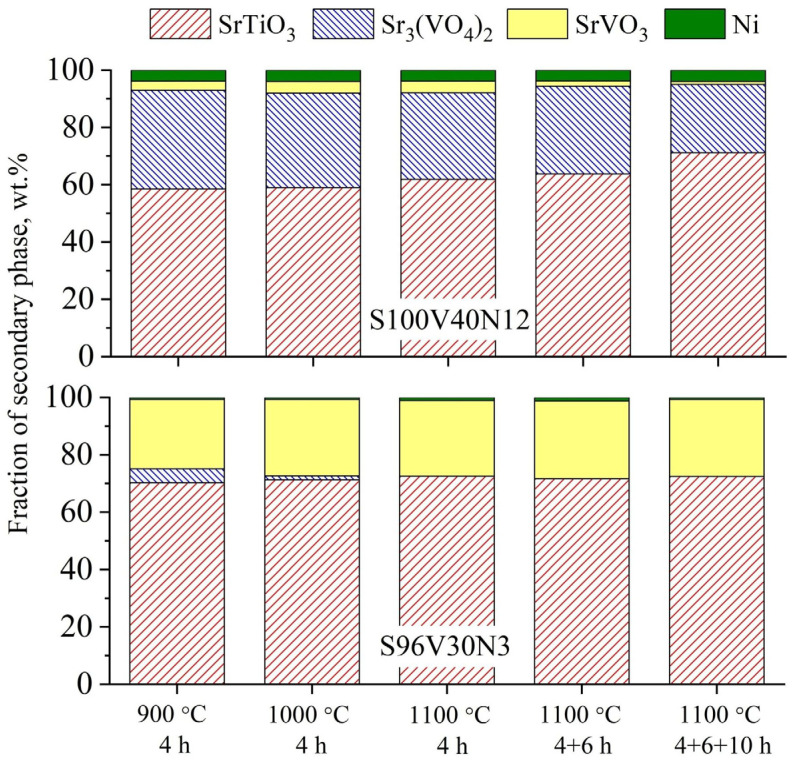
Estimated fractions of different phases in S100V40N12 and S96V30N3 samples after consecutive thermal treatment steps in flowing 10% H_2_–N_2_ at 900–1100 °C.

The estimated fractions of different phases for the two selected compositions (S100V40N12 and S96V30N3) are shown in Figure 2. Corresponding XRD patterns can be found in Figures S5 and S6. The results of thermal treatments at lower temperatures, 800–900 °C, were qualitatively identical to those obtained by the direct calcinations of precursors. In the case of nominally Sr-stoichiometric S100V40N12 with a large Ni content, subsequent firing steps at 1000 °C/4 h and 1100 °C/4 h did not result in a noticeable improvement. The sample still comprised a large fraction of Sr_3_(VO_4_)_2_ (~30–33 wt.%), as well as perovskite-type SrVO_3_ in addition to the main SrTiO_3_-based perovskite phase and metallic nickel. It was noticed that the amount of undesired secondary phases decreased with the increasing calcination time at 1100 °C; however, this occurred very slowly. On the contrary, only a trace amount of Sr_3_(VO_4_)_2_ impurity was detected in the S96V30N3 sample after the thermal treatment step at 1000 °C, and no evidence of this secondary phase was found after subsequent calcination at 1100 °C. Nonetheless, the second perovskite phase was still present even after calcinations at 1100 °C, as suggested by the asymmetry of the reflections of the main perovskite phase (Figure S6); its fraction did not alter with the treatment time (Figure 2).

Thus, consecutive thermal treatment steps did not lead to any noticeable improvements in terms of the formation of the desired phase. This appears to be in agreement with the literature data, which shows that V^5+^-based strontium orthovanadate Sr_3_(VO_4_)_2_ is a stable phase and forms as an intermediate in the course of reduction of oxidized strontium pyrovanadate $Sr_{2}V_{2}^{5+}O_{7}$ to reduced SrV^4+^O_3_ perovskite [16]. Typically, heat treatments at temperatures above 1000 °C are required to eliminate this phase [12,16]. In the present work, the dissolution of the intermediate secondary phases was found to be very slow even at 1100 °C.

#### 3.1.4. Final Synthetic Procedure

Since calcinations at 1100 °C for up to 20 h were still insufficient for the formation of a single Sr(Ti,V)O_3_ perovskite phase combined with metallic nickel, it was decided to conduct solid-state synthesis at a temperature of 1200 °C, which was expected to be low enough to avoid excessive grain growth. Fresh mechanically activated precursors of all compositions were pelletized and fired at 1200 °C for 10 h. XRD inspection of the samples after this thermal treatment revealed that some compositions with a higher nominal Ni content combined with a low or no A-site deficiency (namely, S100V30N6, S100V40N12, and S98V30N9) still contained a non-negligible fraction of Sr_3_(VO_4_)_2_ impurity (Figure S7). These three compositions were excluded from further work. The presence of the second SrVO_3_-based perovskite phase was still suspected at least for one of the remaining compositions (S96V30N3), based on the asymmetry of the XRD reflections. Therefore, six STVN compositions were reground and fired in pelletized form at 1200 °C for an additional 20 h to ensure the full dissolution of secondary oxide phases (Figure S8). These as-synthesized materials were powdered and then used for the preparation of electrode layers and ceramic samples.

### 3.2. Crystal Structure and Microstructure

XRD analysis confirmed that both as-synthesized STVN powders (1200 °C, 30 h) and sintered STVN ceramics (1450 °C, 10 h) comprised a Sr_1-*a*_Ti_1-*b*_V*_b_*O_3-δ_ solid solution with a cubic perovskite-type lattice (space group $Pm\bar{3}m$) isostructural to the parent SrTiO_3_ and SrVO_3_, and a fraction of metallic nickel phase. In agreement with the data on the Sr(Ti,V)O_3-δ_ system [34,48], the unit cell parameter of the perovskite phase shows a tendency to decrease with increasing vanadium content (Table 1), which is consistent with the values of ionic radii for six-fold coordinated V^4+^ and Ti^4+^ (0.58 Å and 0.605 Å, respectively [49]). The only exception to this trend is S96V40N8, the sample with the highest nickel content.

If nickel does not incorporate into the B sublattice of the perovskite structure and segregates completely as the metallic phase, this should result in the cation ratio of Sr:(Ti + V) > 1 for most of the prepared compositions. While strontium vacancies are known to be frequently encountered defects in SrTiO_3_-based solid solutions, the formation of vacancies in the titanium sublattice or the simultaneous formation of strontium and titanium vacancies were shown to be energetically unfavorable [33,50,51,52]. Therefore, excess strontium can be compensated for by precipitation of a secondary Sr-rich phase such as Ruddlesden–Popper Sr_3_Ti_2_O_7_ or strontium orthovanadate Sr_3_(VO_2_)_2_. Given the stability of the latter phase and the difficulties faced during the synthesis, it would be reasonable to assume that traces of the Sr_3_(VO_2_)_2_ phase, undetected by XRD due to their very small amount and dispersed state, may still be present in the samples. In that case, the relationship between the nominal and actual compositions are described by the following equation:

Sr_1-*x*_Ti_1-*y*-*z*_V*_y_*Ni*_z_*O_3_ → *m* Sr_1-*a*_Ti_1-*b*_V*_b_*O_3_ + *n* Sr_3_(VO_4_)_2_ + *z* Ni, (1)

If Equation (1) is true, only one of the synthesized materials actually remains A-site deficient, while the fraction of precipitated Sr_3_(VO_4_)_2_ impurity should correspond to ~2 mol.% in most cases (Table 2).

Most of sintered STVN ceramics were dense, with a relative density between 93 and 97% of the theoretical density (roughly estimated from the structural data assuming the nominal cation composition) (Table 1). The only exception was the S96V30N3 samples which showed a lower relative density of ~89%. The estimated values of relative density are in good agreement with the microstructure of the STVN ceramics observed by SEM (Figure S9). SEM images of fractured S96V30N3 ceramics revealed easily distinguishable individual grains with a size of 0.5–3.5 µm and visible porosity. Other compositions appeared to be comparatively dense, with a minor closed porosity and slightly different microstructural features. The S96V20N6 ceramics were composed of comparatively small grains (1.5–6.0 μm), but their shape implies an onset of formation of denser agglomerates. On the contrary, the SEM of fractured cross-sections of the S98V40N4 and S96V40N8 samples showed dense sintered bodies with mostly indistinguishable individual grains. The microstructure of the S100V20N2 and S98V20N4 samples is an intermediate case comprising both smaller easily distinguishable grains (2–10 μm) and large dense agglomerates with sizes up to 50 µm. Note that SEM/EDS analysis demonstrated a uniform distribution of elements (Sr, Ti, V, and O) in both small grains and larger dense blocks. The changes in microstructural features with composition are caused, apparently, by two main factors. First, increasing vanadium content appears to promote sinterability and grain growth. The second factor is the expected presence of the Sr_3_(VO_4_)_2_ secondary phase. Although the available phase diagram of the SrO-V_2_O_5_ system predicts that Sr_3_(VO_4_)_2_ melts at 1545 °C [53,54], our previous experience of sintering of SrVO_3_-based ceramics [15,16] showed that an excess of Sr_3_(VO_4_)_2_ impurity promotes the melting of the samples already at 1500 °C in 10% H_2_–N_2_ atmosphere even though the melting point of pure SrVO_3_ should be as high as 1950–2000 °C [55,56]. Thus, the traces of Sr_3_(VO_4_)_2_ phase impurity (Table 2) are likely to play the role of sintering aid, promoting the grain growth and densification, while the impurity-free S96V30N3 ceramics remain comparatively porous.

**Figure 3:**
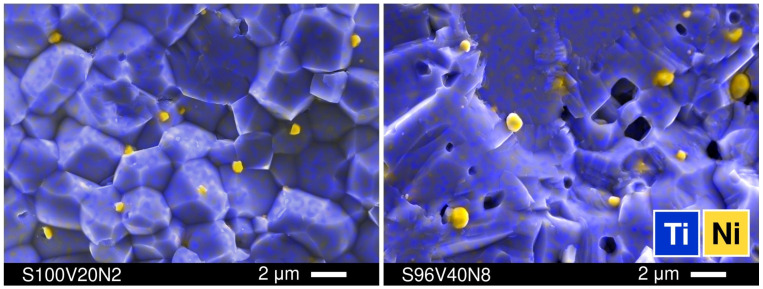
Representative SEM micrographs of the fractured surfaces of S100V20N2 and S96V40N8 ceramics with overlaid EDS elemental mapping.

The distribution of metallic nickel in sintered STVN ceramics was inspected using SEM/EDS. Representative images are shown in Figure 3; the SEM/EDS micrographs for the entire range of compositions can be found in Figure S10. It was observed that the size of the Ni particles correlated with the grain size of the perovskite phase and, therefore, the density of grain boundaries (i.e., the number of available grain boundaries per unit volume). The samples with a denser microstructure and larger grains (e.g., S96V40N8) comprised larger Ni particles, with sizes up to ~1 µm, while the ceramics with smaller well-defined individual grains of the perovskite phase had more evenly distributed smaller metallic Ni inclusions, with sizes varying from 0.2 to 0.7 µm (as in S100V20N2). Note that the easily visible agglomeration of the metallic nickel phase does not completely exclude the possibility of a partial incorporation of Ni cations into the B-sublattice of Sr(Ti,V)O_3-δ_ perovskites.

### 3.3. Thermomechanical Compatibility of STVN with Solid Electrolyte

**Figure 4:**
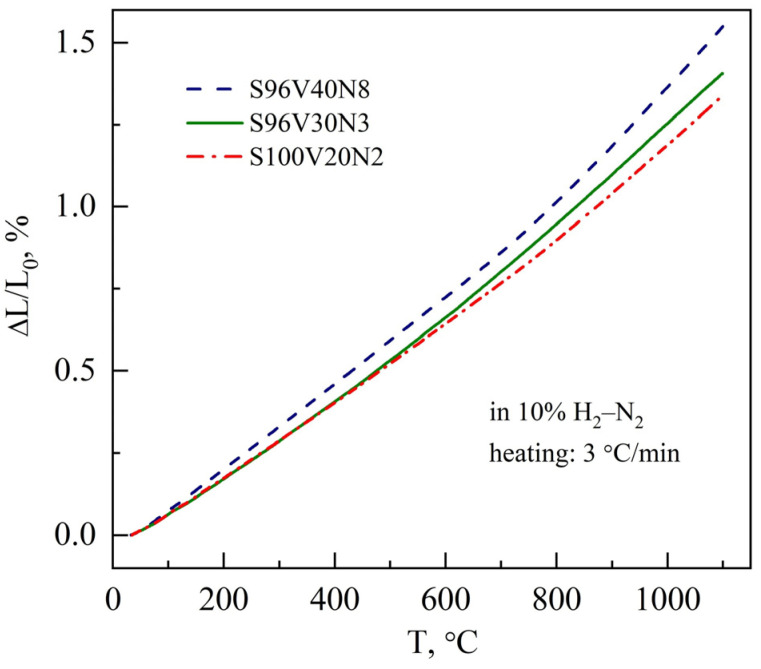
Dilatometric curves of STVN ceramics on heating in a 10% H_2_–N_2_ atmosphere.

The dilatometric curves of the STVN ceramics showed smooth, slightly non-linear behavior upon heating in a reducing 10% H_2_–N_2_ atmosphere. The representative examples for the selected samples are depicted in Figure 4. Similar to SrTi_1-*y*_V*_y_*O_3-δ_ series [34] and other SrVO_3-δ_-based materials [14,15,22], increasing deviation from the linear thermal expansion at higher temperatures can be partly attributed to a minor contribution of chemical expansion associated with the variable oxygen content in the perovskite lattice.

The average linear thermal expansion coefficients (TECs) of the STVN ceramics calculated from the dilatometric data were found to vary in the range of 12.5–14.3 ppm/K at 25–1100 °C, generally decreasing with an increase in titanium content in the perovskite lattice (Table 3). This is consistent with the observation reported earlier for SrVO_3_-SrTiO_3_ solid solutions [34] where increasing titanium content was found to suppress both true thermal expansion and chemical strain and to reduce the average TEC (100–1000 °C) from 19.3 ppm/K for undoped SrVO_3-δ_ to 12.0 ppm/K for titanium-rich SrTi_0.9_V_0.1_O_3-δ_ ceramics.

The TECs of STVN ceramics slightly exceed the corresponding value for the common 8YSZ solid electrolyte ceramics (10.5 ppm/K at 25–1100 °C [57]) but are still very close. This should ensure good thermomechanical compatibility between porous STVN electrodes and 8YSZ solid electrolytes during thermal cycling.

### 3.4. Electrical Conductivity

**Figure 5:**
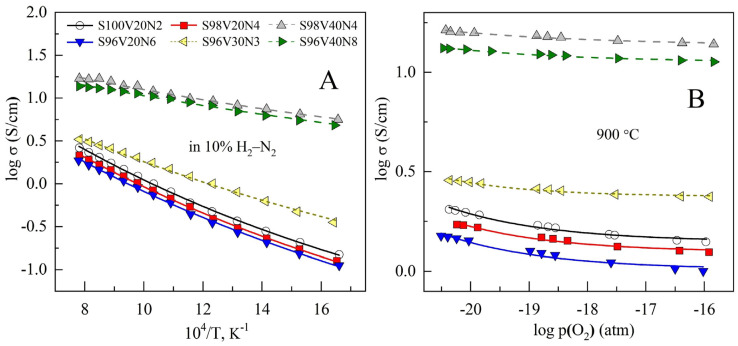
Dependence on the electrical conductivity of STVN ceramics on temperature in 10% H_2_–N_2_ atmosphere (**A**) and on oxygen partial pressure at 900 °C under reducing conditions (**B**).

Sintered STVN ceramics exhibit semiconducting behavior with temperature-activated electrical conductivity under reducing conditions (Figure 5A). Electrical conductivity was found to increase with increasing vanadium content in the perovskite lattice, reaching a maximum of ~17 S/cm at 900 °C for the S98V40Ni4 composition, while the activation energy for conductivity showed an opposite trend (Table 3). These observations are in good agreement with previously reported data on the SrVO_3_-SrTiO_3_ pseudobinary system [34,48]. SrVO_3-δ_ is known to be a metallic conductor with a conductivity level as high as ~10^3^ S/cm at 700–900 °C [12,16]. On the other hand, undoped SrTiO_3-δ_ is a wide-bandgap semiconductor with a conductivity that is ≤10^−1^ S/cm at 900 °C under similar reducing conditions [23,24,25]. Accordingly, decreasing the vanadium content in SrTi_1-*y*_V*_y_*O_3-δ_ solid solutions was demonstrated to result in a gradual decrease in electrical conductivity and a transition from metallic to semiconducting behavior [34,48]. This was interpreted in terms of a gradual change in the mechanism of electronic conduction.

The charged point defects in Sr_1-*x*_Ti_1-*y*_V*_y_*O_3-δ_ solid solutions based on the Sr^2+^Ti^4+^O^2−^ host lattice include vacancies in the strontium sublattice, Ti^3+^ and V^3+^ cations in the B-sublattice, and vacancies in the oxygen sublattice. The corresponding electroneutrality condition is given by (using Kröger–Vink notation):

(2) $$2\left[{⎕}_{\mathrm{Sr}}^{\prime \prime }\right]+\left[{\mathrm{Ti}}_{\mathrm{Ti}}^{\prime }\right]+\left[V_{\mathrm{Ti}}^{\prime }\right]=2\left[{⎕}_{O}^{••}\right],$$

where ⎕ indicates a vacancy, ${\mathrm{Ti}}_{\mathrm{Ti}}^{\prime }\equiv {\mathrm{Ti}}^{3+}$, and $V_{\mathrm{Ti}}^{\prime }\equiv V^{3+}$. It is expected that the electronic transport in the compositions with a vanadium content of ~20–40 at.% in the titanium sublattice is carried out by the electrons localized on the titanium cations and is contributed to by electron hopping between V^4+^/V^3+^ pairs [34]. Thus, the concentration of electronic charge carriers is equivalent to the concentration of Ti^3+^ and Vi^3+^. As vanadium cations are more easily reducible, the electronic transport via V^4+^/V^3+^ redox pairs should prevail and declines when the total vanadium content decreases. The variations in the conductivity of the samples with a similar vanadium content in the perovskite lattice can be attributed to an interplay between the porosity, microstructural features, and the distribution of insulating Sr_3_(VO_4_)_2_ impurity.

Reducing oxygen partial pressure results in a modest increase in the conductivity for all STVN compositions (Figure 5B). This should be attributed to an increase in the concentration of *n*-type electronic charge carriers due to the reduction of B-site transition metal cations and oxygen release from the lattice:

(3) $$2B_{\mathrm{Ti}}^{\times }+O_{O}^{\times }\to 2B_{\mathrm{Ti}}^{\prime }+\left[{⎕}_{O}^{••}\right]+0.5O_{2},$$

where B is Ti or V. The dependence of conductivity on p(O_2_) is very weak and resembles that reported for titanium-rich SrTi_1-*y*_V*_y_*O_3-δ_ solid solutions under similar conditions [34].

The electrical conductivity of electrode materials for SOFC/SOEC should be as high as possible to minimize ohmic losses and avoid limiting effects on electrochemical activity. It is generally considered that the minimum required electronic conductivity corresponds to >1 S/cm for porous electrode structures and one order of magnitude higher value for the bulk materials [58]. The conductivity of the STVN ceramics with the highest vanadium content, S98V40N4 and S96V40N8, amounts to 10–17 S/cm at 700–900 °C and, thus, appears to be acceptable for electrode application. Other STVN materials exhibit 5–12 times lower electrical conductivity in the same temperature range. Hence, comparatively low conductivity may be one of the performance-limiting factors in the case of electrode structures based on these compositions.

### 3.5. Electrochemical Characterization of STVN Electrodes

#### 3.5.1. As-Prepared STVN Electrodes

**Figure 6:**
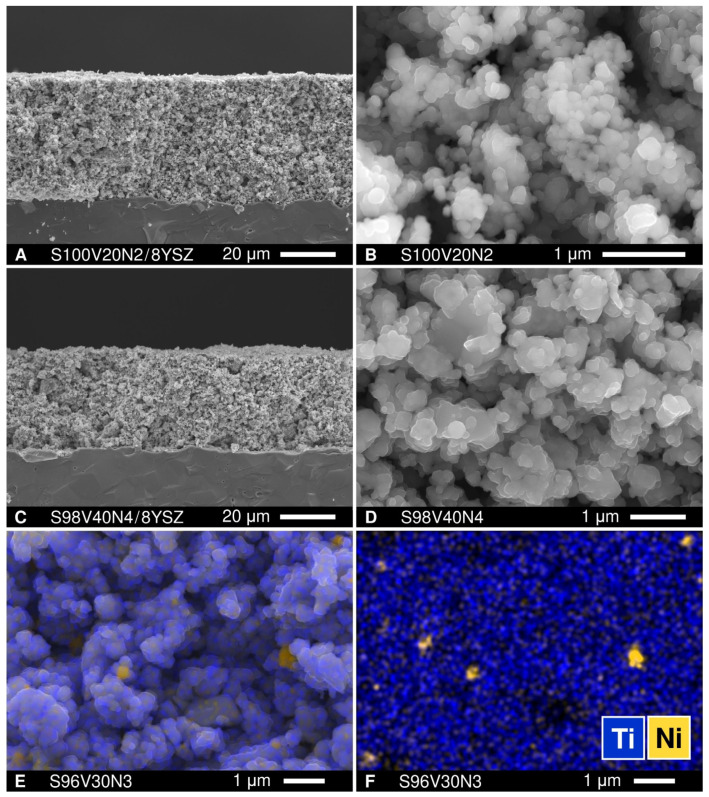
(**A**,**C**) SEM micrographs of fractured cross-sections of SVTN|8YSZ assemblies; (**B**,**D**) enlarged views of the microstructures of porous S100V20N2 and S98V40N4 electrodes; (**E**) SEM micrograph of the S96V30N3 electrode with overlaid EDS elemental mapping; and (**F**) corresponding EDS elemental mapping shown separately for clarity.

The initial electrochemical experiments aimed at a comparative assessment of the activity of plain as-prepared STVN porous electrode layers applied onto 8YSZ solid electrolytes using a symmetrical electrode|electrolyte|electrode cell configuration. Figure 6 shows some representative images illustrating the microstructures of the electrodes. The porous electrode layers with a thickness of approximately 35–45 μm consisted mainly of submicron STVN particles (0.1–0.6 μm) combined with occasional agglomerates with sizes of up to ~1.5 μm. Further inspection using SEM/EDS revealed the presence of agglomerated Ni particles with a diameter of 0.25–0.60 μm. The possible presence of smaller Ni particles distributed along the surface of bare STVN electrodes could not be verified with the resolution of the employed SEM/EDS equipment.

**Figure 7:**
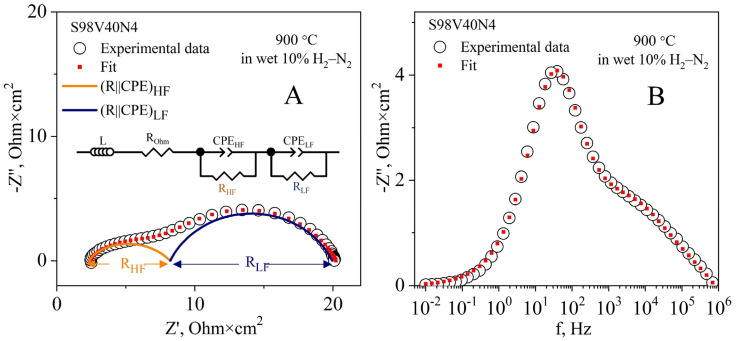
A representative impedance spectrum of a symmetrical S98V40N4|8YSZ|S98V40N4 cell in humidified 10% H_2_–N_2_ at 900 °C: Nyquist (**A**) and Bode (**B**) plots. Open and closed circle symbols correspond to the experimental data and the results of fitting, respectively; the corresponding equivalent circuit is shown as an inset in (**A**). High- and low-frequency (HF and LF) contributions are represented by orange and dark blue semicircles, respectively.

A typical impedance spectrum of a symmetrical cell with STVN electrodes is presented in Figure 7A (Nyquist plot) and Figure 7B (Bode plot). All collected spectra were satisfactorily fitted to a simple equivalent circuit (inset in Figure 7A) comprising an inductive element (L), the ohmic resistance of the cell (R_Ohm_), and two (R||CPE) contributions (where R is resistance and CPE is a constant phase element [59,60]) at high and low frequencies (HF and LF, respectively) corresponding to the electrode processes. The sum of R_HF_ and R_LF_ provides the specific electrode polarization resistance (R_p_). Taking into account the literature data (e.g., Refs. [61,62]), the HF contribution (frequencies in the order of kHz) is presumably associated with the ionic/electronic charge transfer at the triple-phase boundary (TPB), whereas LF components (frequencies below 1 kHz) are generally ascribed to the gas kinetics at the electrode surface (non-Faradaic mass transport mechanisms such as hydrogen adsorption/desorption and surface diffusion of active species).

**Figure 8:**
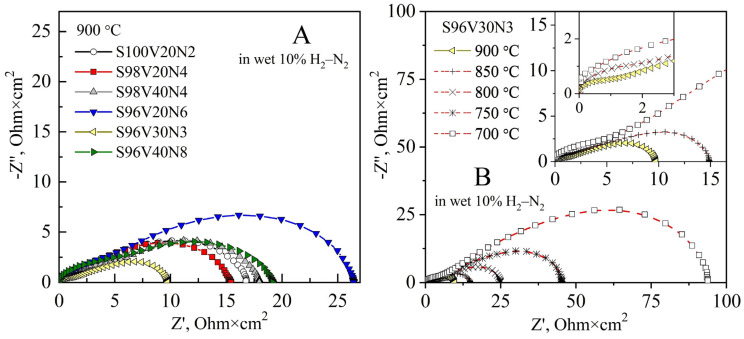
(**A**) Comparison of impedance spectra of symmetrical STVN|8YSZ|STVN cells in humidified 10% H_2_–N_2_ at 900 °C. (**B**) Evolution of impedance spectra of a symmetrical S96V30N3|8YSZ|S96V30N3 cell between 700 and 900 °C. All spectra are corrected for the ohmic contribution.

**Figure 9:**
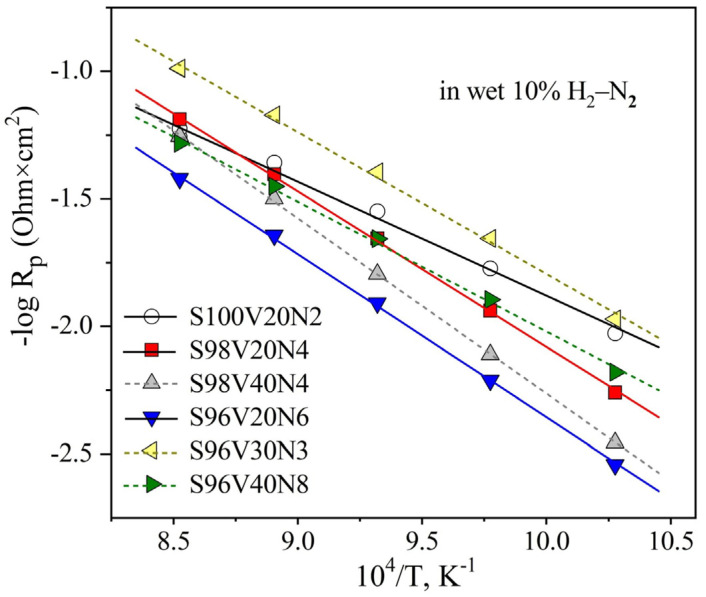
Temperature dependence of the area-specific electrode polarization resistance R_p_ of as-prepared STVN electrodes in humidified 10% H_2_–N_2_.

The STVN electrodes were found to exhibit rather poor electrocatalytic activity for hydrogen oxidation reaction even at 900 °C, with R_p_ values varying between 9.8 and 26.4 Ohm × cm^2^ (Figure 8A). In all cases, the LF contribution was the dominant performance-limiting process. Decreasing temperature results in slower kinetics of the electrode process, with both HF and LF contributions exponentially increasing upon cooling. Figure 8B illustrates the evolution of EIS spectra between 700 and 900 °C for the S96V30N3-based cells, with the R_p_ value increasing from 9.8 to 94 Ohm × cm^2^ upon cooling. The temperature dependence of electrode polarization resistance is depicted in Figure 9. The S96V30N3 and S96V20N6 electrodes exhibit the best and the worst performance, respectively, in the entire temperature range. At the same time, the values of E_A_ vary in a wide range from 95 kJ/mol (S100V20N2) to 141 kJ/mol (S98V40N4).

The analysis of the results did not reveal any evident correlation between the nominal nickel content in the STVN, the electrical conductivity of ceramics, electrode polarization resistance, and the corresponding activation energy. Therefore, one may conclude that the unsatisfactory electrochemical performance of the as-prepared STVN electrodes can be attributed to the poor intrinsic electrocatalytic activity of Sr_1-*x*_Ti_1-*y*_V*_y_*O_3-δ_ perovskites in combination with a low expected ionic conductivity in these phases. The high firing temperature required to eliminate an undesired insulating Sr_3_(VO_4_)_2_ impurity phase promoted the agglomeration of catalytically active Ni particles. Therefore, it can be assumed that the differences in the obtained R_p_ values for different STVN electrodes are mainly caused by a somewhat different uncontrolled distribution of the segregated nickel phase.

#### 3.5.2. Chemical Reactivity between STVN and Solid Electrolytes

**Figure 10:**
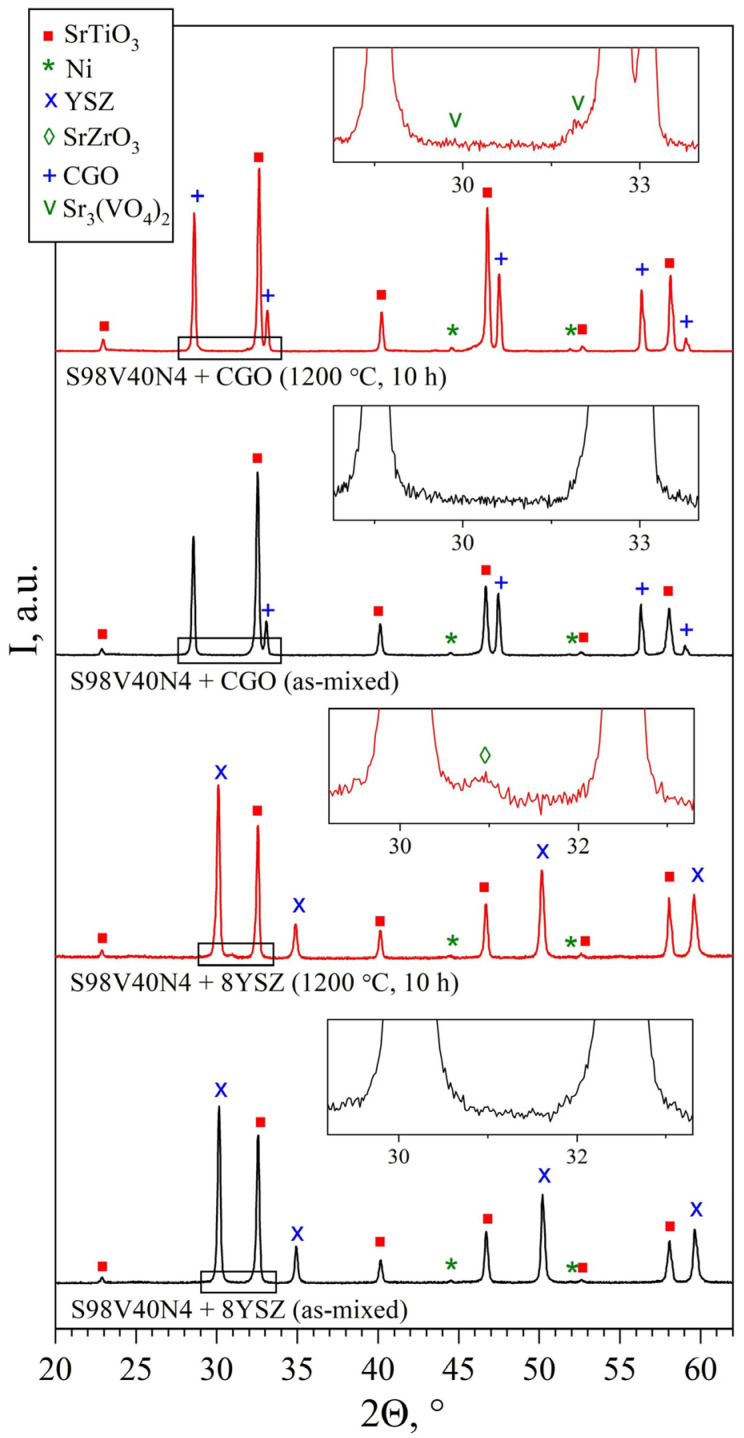
XRD patterns of S98V40N4 + 8YSZ and S98V40N4 + CGO powder mixtures (50:50 wt.%): as-mixed and after calcination in flowing 10% H_2_–N_2_ at 1200 °C for 10 h.

The reactivity between the electrode and the electrolyte material during the fabrication and/or operation of solid oxide cells is one of the factors that may have a negative impact on the overall performance of SOFCs. The undesired cation interdiffusion may result in the formation of insulating products at the electrode/electrolyte interface, blocking the mass-transfer processes (oxygen diffusion). In the present study, the SEM/EDS inspection of the electrode/electrolyte assemblies after fabrication and after experiments did not reveal a formation of any interlayers at the STVN/8YSZ interfaces. Since the thickness of such layers may be too low for the resolution of the SEM/EDS, additional reactivity tests were conducted. Powdered STVN + 8YSZ mixtures (50:50 wt.%) were fired at 1200 °C for 10 h in a flowing 10% H_2_–N_2_ atmosphere and then analyzed using XRD. The appearance of a very small additional peak at 2Θ ≈ 30.9° was detected in the XRD patterns after calcination (Figure 10). This peak can be assigned to the perovskite-like SrZrO_3_ phase, a poorly conducting reactivity product that is frequently observed at the (La,Sr)MnO_3_/8YSZ interface and has a negative effect on the performance of (La,Sr)MnO_3_-based cathodes in SOFCs [63]. Nonetheless, the reactivity between 8YSZ and STVN under applied firing conditions was found to be very limited. As the actual STVN electrode layers in the present work were sintered at a lower temperature (1100 °C) and for a shorter time (2 h), one may assume a rather limited adverse impact of possible reactivity between the materials on the electrode performance.

A common practice to improve the electrochemical activity of electrode materials with predominant electronic conductivity and poor ionic transport is to introduce a fraction of solid electrolyte material into the porous electrode layer, thus forming a composite electrode. This results in a drastic increase in the triple-phase boundary length (i.e., the concentration of sites where the electrochemical reaction takes place) and, correspondingly, an improvement in electrochemical activity. As gadolinia-doped ceria (CGO) is often used for this purpose, the reactivity between CGO (Ce_0.9_Gd_0.1_O_2-δ_ in the present work) and STVN was tested in a similar manner as in the case for 8YSZ. A careful inspection of the XRD patterns of the STVN + CGO mixtures (50:50 wt.%) after calcination at 1200 °C for 10 h in 10% H_2_–N_2_ atmosphere revealed the presence of several additional very small peaks on the background level (Figure 10). These tiny peaks coincided with the positions of the main reflections of the Sr_3_(VO_4_)_2_ phase. As discussed above, the traces of this phase are expected to be present in the synthesized STVN to compensate for the segregation of metallic nickel. It is possible that a partial reduction of CGO at 1200 °C accompanied by the release of oxygen from the fluorite lattice may promote the transformation of an additional fraction of Sr(Ti,V)O_3-δ_ perovskite into the oxidized strontium orthovanadate phase. However, no evidence of chemical reactivity between STVN and CGO with the formation of additional phases could be detected at temperatures as high as 1200 °C.

#### 3.5.3. Modification of STVN Electrodes via Infiltration

**Figure 11:**
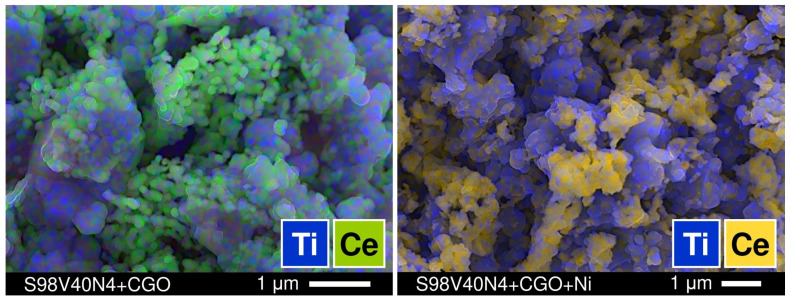
SEM micrographs with overlaid EDS elemental mapping illustrating the microstructure of modified electrodes and distribution of STVN and CGO phases. (**left**) S98V40N4 electrode infiltrated with CGO (27 wt.%); (**right**) S98V40N4 electrode infiltrated with CGO and Ni (30 wt.%, CGO:Ni = 10:1).

As plain STVN electrodes demonstrated poor electrochemical activity, the modification of porous electrode layers through the incorporation of additional components—CGO as the oxygen-ion-conducting phase and nano-sized Ni as the electrocatalyst—was attempted in order to improve their performance. Two STVN compositions were selected for these studies: one with the best performance in the unmodified form (S96V30N3) and one with the average performance (S98V40N4). The modification was completed via the infiltration procedure described in Section 2.4. Three combinations of additional components were tested including (i) only CGO, (ii) CGO + Ni with a molar ratio 10:1, and (iii) CGO + Ni with a molar ratio 2:1. The total load of infiltrated components after thermal treatment corresponded to 21–30 wt.%. This provides the fraction of extra Ni catalyst equal to ~1 and ~4 wt.% in the case of the CGO:Ni ratio of 10:1 and 2:1, respectively. The SEM/EDS inspection of modified electrodes revealed that the porous STVN backbone was uniformly covered by submicron CGO particles (100–150 nm) and well-dispersed nanostructured Ni catalysts (Figure 11, Figures S11 and S12).

**Figure 12:**
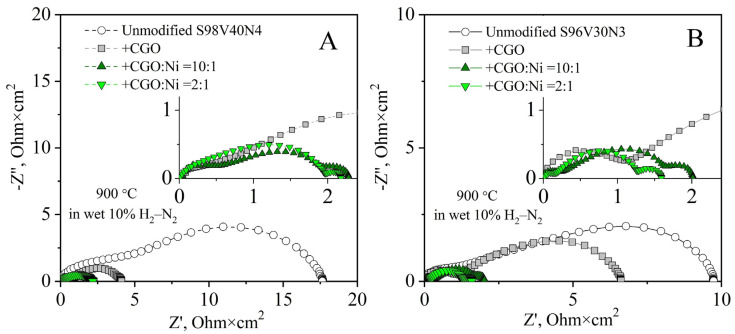
Impedance spectra of as-prepared (unmodified) and infiltrated S98V40N4 (**A**) and S96V30N3 (**B**) electrodes in humidified 10% H_2_–N_2_ at 900 °C. All spectra are corrected for ohmic contributions. Insets show magnified sections of the plots.

The incorporation of CGO (~21 wt.%) as a solid electrolyte component resulted in a reduction in electrode polarization resistance (Figure 12). While this change was moderate in the case of the S96V30N3-based cell, from 9.8 to 6.6 Ohm × cm^2^ at 900 °C, the R_p_ of the S98V40N4 electrodes dropped more than four times from ~18 to 4.2 Ohm × cm^2^ at 900 °C. The enhanced performance is attributed to an increase in the TPB length as a result of the introduction of the oxygen-ion conducting phase into the electrode structure.

Co-infiltration of CGO and Ni (27–30 wt.%, CGO:Ni = 10:1) substantially improved the electrochemical activity of STVN electrodes; the R_p_ values dropped 5–7 times compared with the as-prepared electrodes. Again, this enhancement was stronger for the S98V40N4-based cell, where electrode polarization resistance decreased to 2.3 Ohm × cm^2^ at 900 °C. Compared with the electrodes infiltrated only with CGO, the decrease in R_p_ values for electrodes co-modified with CGO + Ni is mainly associated with the reduced LF contribution. This implies that the addition of a dispersed Ni catalyst leads to enhanced kinetics of processes at the electrode/gas interface.

Further increasing the fraction of infiltrated nickel (CGO:Ni = 2:1) was found to have a comparatively small effect on the electrode polarization resistance of S96V30N3-based electrodes (R_p_ = 1.6 Ohm × cm^2^ at 900 °C) or even a slightly negative impact in the case of S98V40N4 (2.6 Ohm × cm^2^ at 900 °C). This seems to indicate that an increased Ni load promotes the agglomeration of Ni particles rather than their better distribution. As a result, the concentration of catalytically active sites and the electrode kinetics are not further improved. Another observation is the emergence of an additional contribution at low frequencies (f < 3 Hz) in the impedance spectra of the infiltrated cells. According to the literature, this contribution can be assigned to the gas diffusion processes. As these processes are mainly affected by the electrode microstructure, the magnitude of this contribution may vary from one electrode to another and does not depend on temperature [64]. In the present case, it becomes visible in the spectra when the overall polarization resistance decreases and the infiltrated components reduce the porosity of the electrode layer, apparently introducing gas-diffusion limitations.

**Figure 13:**
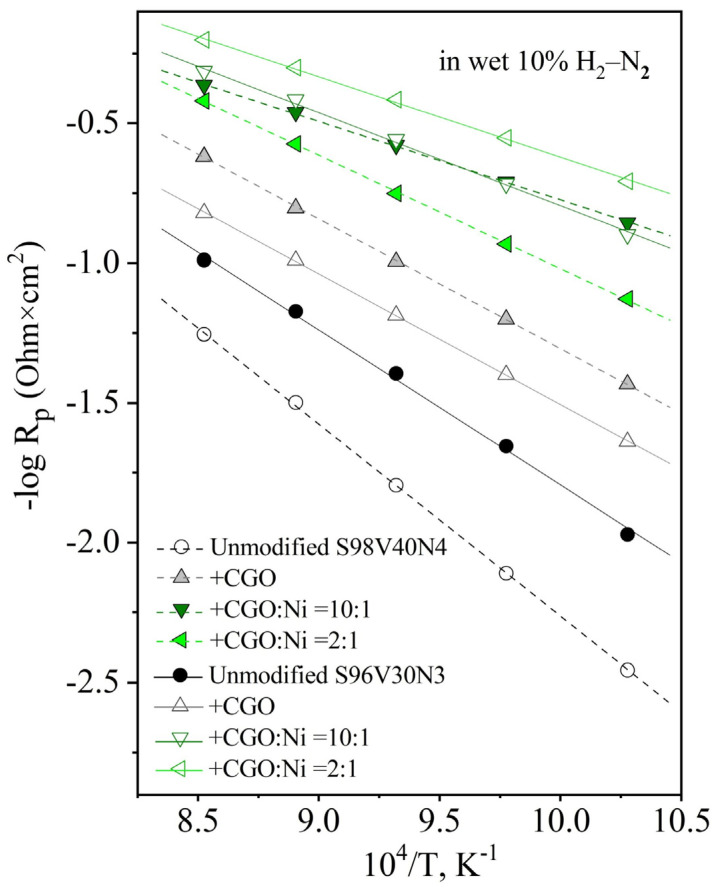
Temperature dependence of the area-specific electrode polarization resistance R_p_ of unmodified and infiltrated S98V40N4 and S96V30N3 electrodes in humidified 10% H_2_–N_2_.

Figure 13 presents the temperature dependence of electrode polarization resistance of unmodified and infiltrated electrodes. The incorporation of CGO or CGO in combination with Ni gradually decreases the activation energy of the electrode process (Table 4), thus leading to a stronger relative improvement in overall electrode performance at lower temperatures. Interestingly, both E_A_ and R_p_ values for S98V40N4 and S96V30N3 co-infiltrated with CGO and Ni (ratio 10:1) become similar. This seems to confirm that the porous STVN skeleton plays a rather passive role in terms of the electrocatalytic activity of infiltrated electrode layers.

## 4. Conclusions

A series of strontium titanate-vanadate (STVN) solid solutions with the nominal cation composition of Sr_1-*x*_Ti_1-*y*-*z*_V*_y_*Ni*_z_*O_3-δ_ (*x* = 0–0.04, *y* = 0.20–0.40 and *z* = 0.02–0.12) were prepared by the solid-state reaction route in reducing 10% H_2_–N_2_ atmosphere. High-energy mechanochemical pre-treatments of the precursor mixtures have a weak effect in terms of the formation of the target perovskite phase. Prolonged firing at 1200 °C is required to eliminate the highly stable Sr_3_(VO_4_)_2_ intermediate phase. Under such conditions, Ni tends to segregate as a metallic phase and is unlikely to incorporate into the perovskite structure. The electrical conductivity of ceramics sintered at 1500 °C is temperature-activated and increases with increasing vanadium content reaching a maximum of ~17 S/cm at 1000 °C. The prepared STVN materials show good thermomechanical and chemical compatibility with 8YSZ and CGO solid electrolytes. Porous STVN electrodes applied onto 8YSZ solid electrolytes demonstrate rather poor electrochemical activity in diluted H_2_. This is attributed to the low intrinsic electrocatalytic activity and low ionic conductivity of Sr(Ti,V)O_3-δ_ perovskites as well as the agglomeration of metallic Ni during the synthetic procedure. The overall performance of STVN electrodes can be substantially improved by the additions of CGO as an oxygen-ion conducting phase (20–30 wt.%) and surface modification by nano-sized nickel as an electrocatalyst (≥1 wt.%).

The preparation of single-phase Sr_1-*x*_Ti_1-*y*-*z*_V*_y_*Ni*_z_*O_3-δ_ perovskites and fabrication of Sr(Ti,V)O_3_-based electrodes decorated with a nanostructured Ni catalyst via an exsolution process requires the development of an alternative synthetic procedure which will make it possible to eliminate or avoid the formation of undesired phases. This is planned as a continuation of the present work.

## Figures and Tables

**Table 1:** Nominal cation composition, perovskite lattice parameters, and density of Sr_1-*α*_Ti_1-*β*(1+γ)_V*_β_*Ni*_βγ_*O_3-δ_ (STVN) ceramics.

α	β	γ	Nominal Composition	Notation	Lattice Parameter *a*, Å	Density ρ_exp_, g/cm^3^	Relative Density ρ_exp_/ρ_theor_, % ^1^
0	0.2	0.1	SrTi_0.78_V_0.20_Ni_0.02_O_3-δ_	S100V20N2	3.9000(1)	4.93	95.5
0	0.3	0.2	SrTi_0.64_V_0.30_Ni_0.06_O_3-δ_	S100V30N6	-	-	-
0	0.4	0.3	SrTi_0.48_V_0.40_Ni_0.12_O_3-δ_	S100V40N12	-	-	-
0.02	0.2	0.2	Sr_0.98_Ti_0.76_V_0.20_Ni_0.04_O_3-δ_	S98V20N4	3.9006 (1)	4.91	95.9
0.02	0.3	0.3	Sr_0.98_Ti_0.61_V_0.30_Ni_0.09_O_3-δ_	S98V30N9	-	-	-
0.02	0.4	0.1	Sr_0.98_Ti_0.56_V_0.40_Ni_0.04_O_3-δ_	S98V40N4	3.8868 (2)	4.89	94.4
0.04	0.2	0.3	Sr_0.96_Ti_0.74_V_0.20_Ni_0.06_O_3-δ_	S96V20N6	3.9018 (1)	4.70	92.7
0.04	0.3	0.1	Sr_0.96_Ti_0.67_V_0.30_Ni_0.03_O_3-δ_	S96V30N3	3.8956 (1)	4.53	88.9
0.04	0.4	0.2	Sr_0.96_Ti_0.52_V_0.40_Ni_0.08_O_3-δ_	S96V40N8	3.9024 (1)	4.96	97.4

^1^ ρ_theor_ was estimated from the structural data assuming nominal cation composition and stoichiometric oxygen content.

**Table 2:** Composition of the samples recalculated according to Equation (1).

Notation	Perovskite Phase	Fraction of Sr_3_(VO_4_)_2_, mol.% ^1^
S100V20N2	SrTi_0.830_V_0.170_O_3_	2.04
S98V20N4	SrTi_0.826_V_0.174_O_3_	2.04
S98V40N4	SrTi_0.609_V_0.391_O_3_	2.04
S96V20N6	SrTi_0.822_V_0.178_O_3_	2.05
S96V30N3	Sr_0.990_Ti_0.691_V_0.309_O_3_	0
S96V40N8	SrTi_0.619_V_0.381_O_3_	4.20

^1^ n/(m + n + z).

**Table 3:** Average thermal expansion coefficients and parameters of the Arrhenius model for the electrical conductivity of STVN ceramics in a 10%H_2_–N_2_ atmosphere.

Composition	Average TEC ± 0.1, ppm/K (25–1100 °C)	Parameters of Arrhenius Model for σ (500–1000 °C) ^1^	
		E_A_, kJ/mol	ln(A_0_) (S/cm)
S100V20N2	12.5	31.4 ± 0.3	3.91 ± 0.03
S98V20N4	12.6	30.8 ± 0.2	3.65 ± 0.02
S98V40N4	14.0	11.3 ± 0.1	3.90 ± 0.02
S96V20N6	13.0	30.6 ± 0.3	3.50 ± 0.03
S96V30N3	13.3	22.4 ± 0.1	3.33 ± 0.02
S96V40N8	14.3	10.0 ± 0.1	3.58 ± 0.02

^1^ σ = A_0_ × exp(−E_A_/(RT)) where E_A_ is the activation energy and A_0_ is the pre-exponential factor.

**Table 4:** Activation energy E_A_ of the electrode process for symmetrical cells with as-prepared (unmodified) and infiltrated STVN electrodes in humidified 10%H_2_–N_2_ atmosphere at 700–900 °C.

Electrode	S98V40N4		S96V30N3	
	Load, wt.%	E_A_, kJ/mol ^1^	Load, wt.%	E_A_, kJ/mol
Unmodified	-	141 ± 1	-	116 ± 3
+ CGO	21	97 ± 1	21	99 ± 1
+ CGO:Ni = 9:1	30	63 ± 1	27	73 ± 2
+ CGO:Ni = 5:1	27	87 ± 1	21	64 ± 1

^1^ calculated using the Arrhenius model (1/R_p_) = (A_0_/T) × exp(−E_A_/(RT)) where A_0_ is the pre-exponential factor.

## Data Availability

Data is contained within the article and/or available from the corresponding author upon reasonable request.

## Supplementary Materials

The following are available online at https://www.mdpi.com/article/10.3390/ma15010278/s1: Table S1: Taguchi planning matrix used for the selection of compositions; Figures S1–S8: additional XRD data; and Figures S9–S12: additional SEM images and SEM/EDS data.
