# Supplementary material for: Development of Ni-Sr(V,Ti)O3-δ Fuel Electrodes for Solid Oxide Fuel Cells
Source: Materials (Basel). 2021 Dec 30;15(1):278. doi: 10.3390/ma15010278 (PMC8746223; doi:10.3390/ma15010278)
Supplement: Supplementary file 1 [file materials-15-00278-s001.zip › materials-1509568-supplementary.pdf]

Supplementary Materials

Development of Ni-Sr(V,Ti)$O_{3-\delta}$ Fuel Electrodes for Solid Oxide Fuel Cells

Bernardo F. Serôdio Costa [1], Blanca I. Arias-Serrano [1,2,*] and Aleksey A. Yaremchenko [1,*]

[1] Department of Materials and Ceramic Engineering, CICECO—Aveiro Institute of Materials, University of Aveiro, 3810-193 Aveiro, Portugal; bernardo.costa.90@gmail.com
[2] Leibniz Institute for Plasma Science and Technology, Felix-Hausdorff-Str. 2, 17489 Greifswald, Germany
* Correspondence: arias-serrano@inp-greifswald.de (B.I.A.-S.); ayaremchenko@ua.pt (A.A.Y.)

Table S1. Taguchi planning matrix for cation compositions of $Sr_{1-\alpha}Ti_{1-\beta(1+\gamma)}V_{\beta}Ni_{\beta\gamma}O_{3-\delta}$ (STVN) series.

**Level, *n***	**Variable, *v***		
	α	*β*	*γ*
1	0	0.2	0.1
2	0.02	0.3	0.2
3	0.04	0.4	0.3

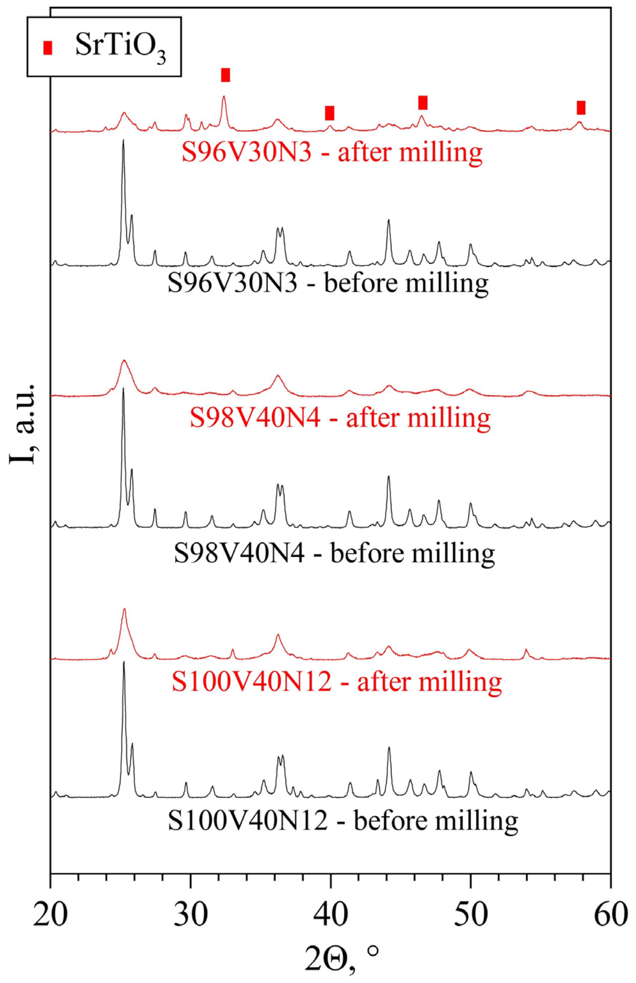

Figure S1. XRD patterns of selected precursor mixtures before and after the mechanical activation. An onset of target $SrTiO_3$-based perovskite phase was observed for S96V30N3 as a result of high-energy mechanical treatment.

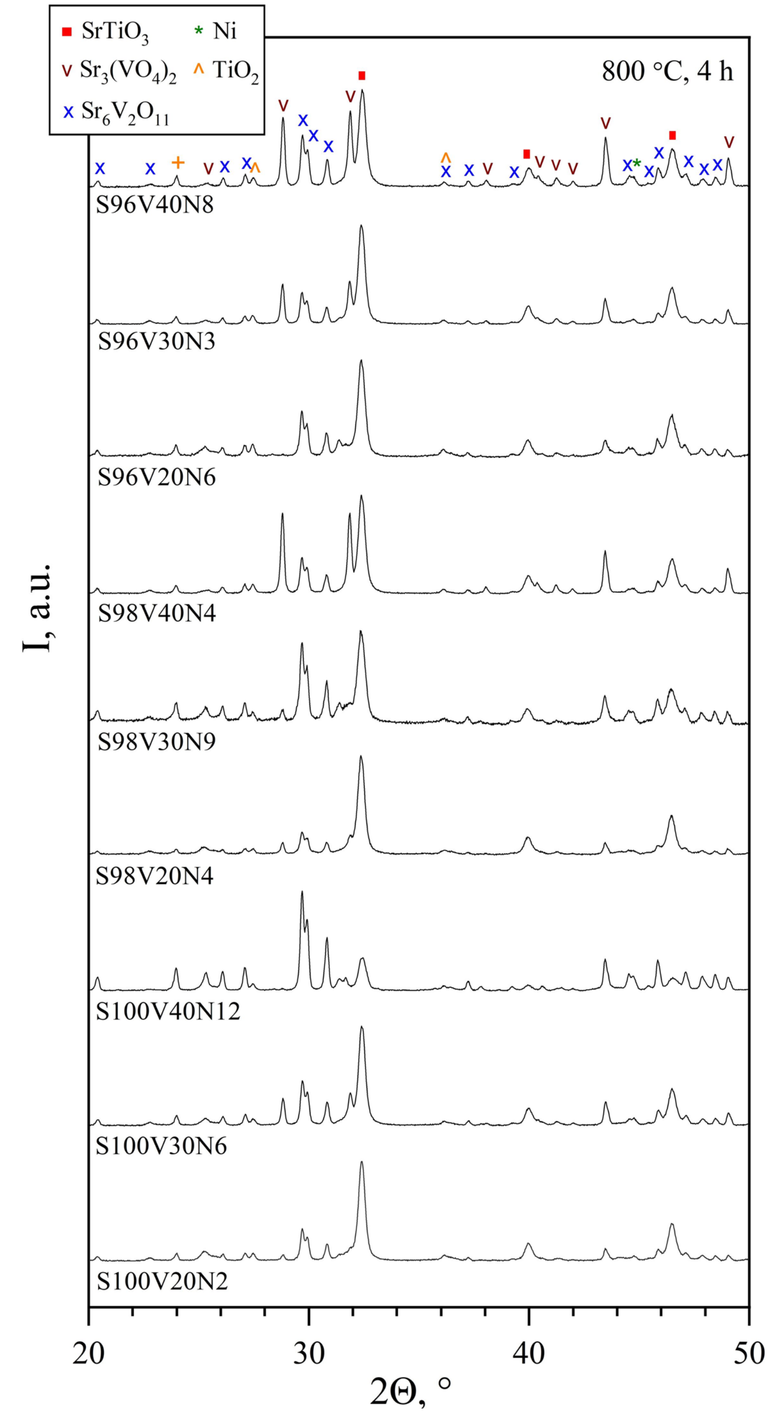

Figure S2. XRD patterns of SVTN samples after calcination for 4 h in flowing 10%H_2-N_2 atmosphere at 800 °C. Phase identification is representatively shown for the S96V40N8 sample.

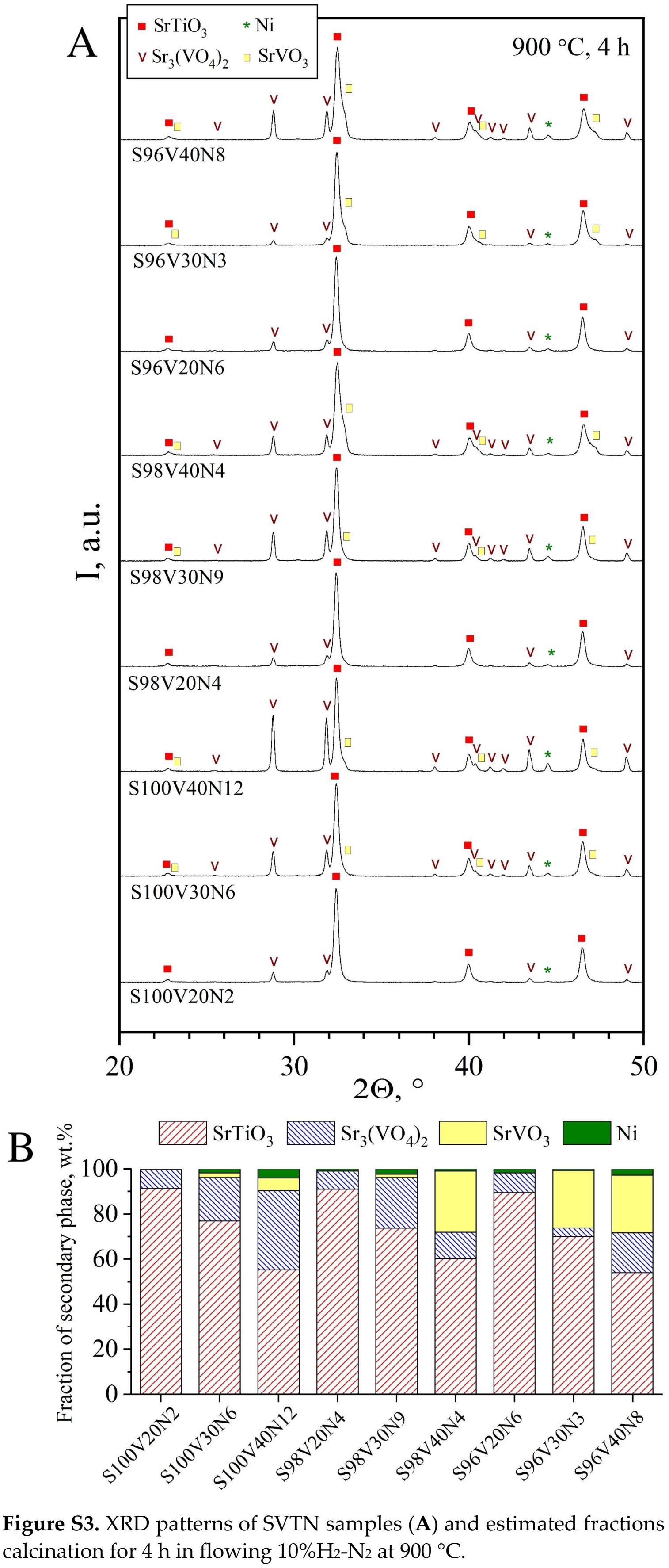

Figure S3. XRD patterns of SVTN samples (**A**) and estimated fractions of different phases (**B**) after calcination for 4 h in flowing 10%H_2-N_2 at 900 °C.

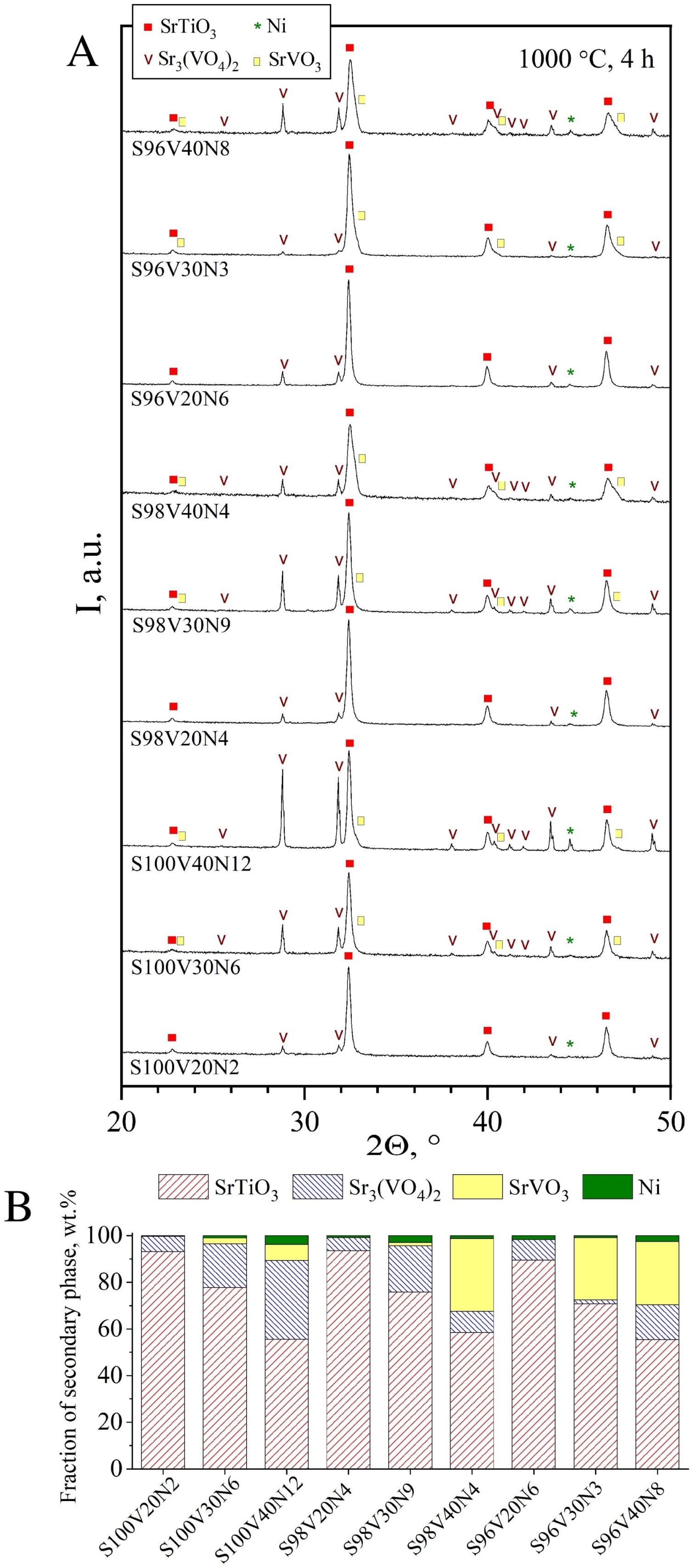

Figure S4. XRD patterns of SVTN samples (**A**) and estimated fractions of different phases (**B**) after calcination for 4 h in flowing 10%H_2-N_2 at 1000 °C.

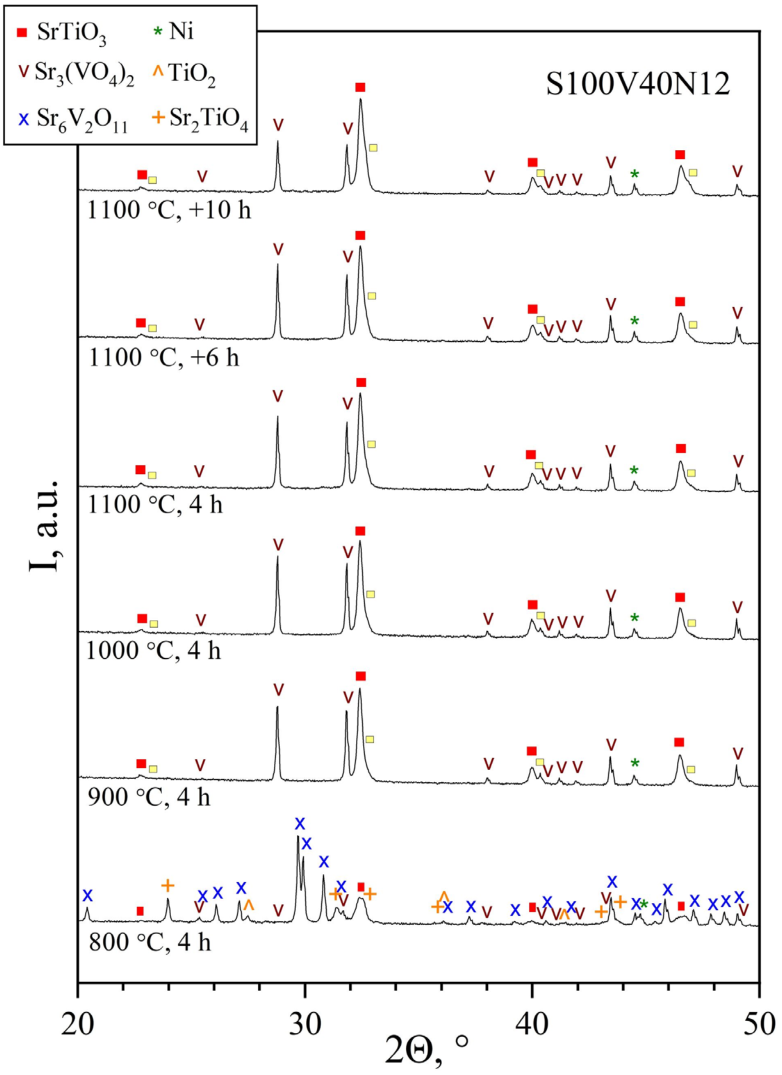

Figure S5. XRD patterns of SV40N12 sample after consecutive thermal treatment steps in flowing 10%H_2-N_2 at 800-1100 °C.

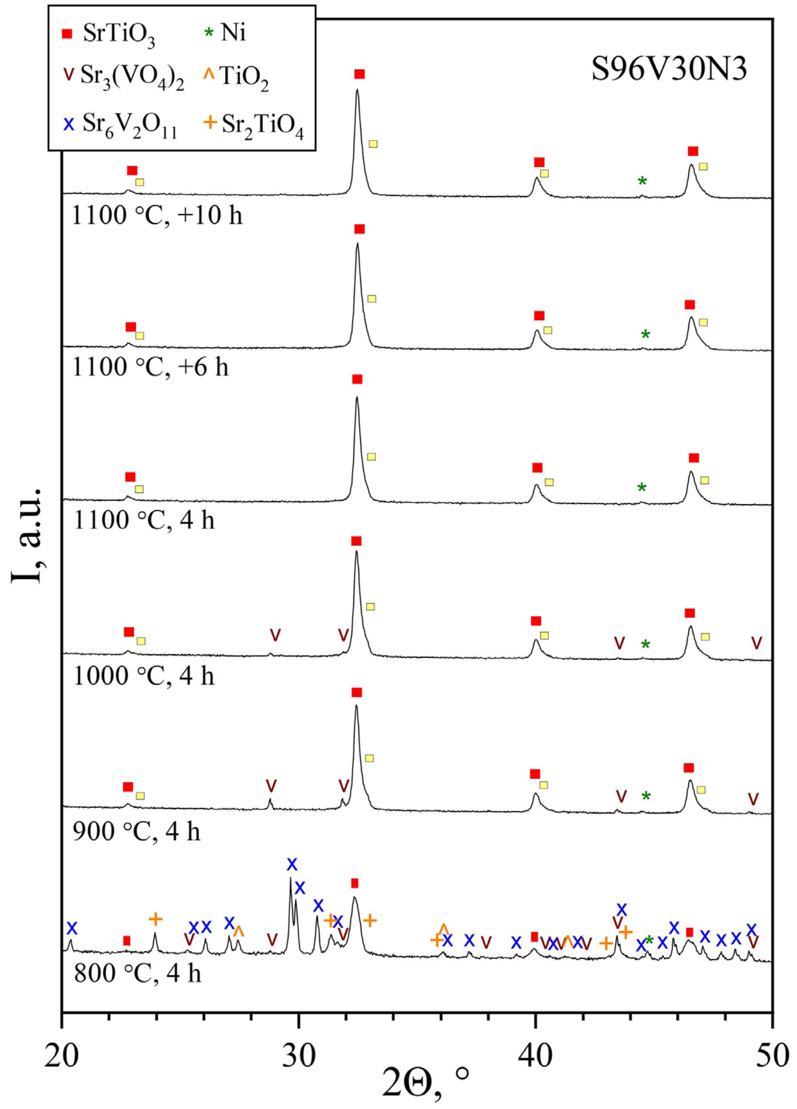

Figure S6. XRD patterns of S96V30N3 sample after consecutive thermal treatment steps in flowing 10%H_2-N_2 at 800-1100 °C.

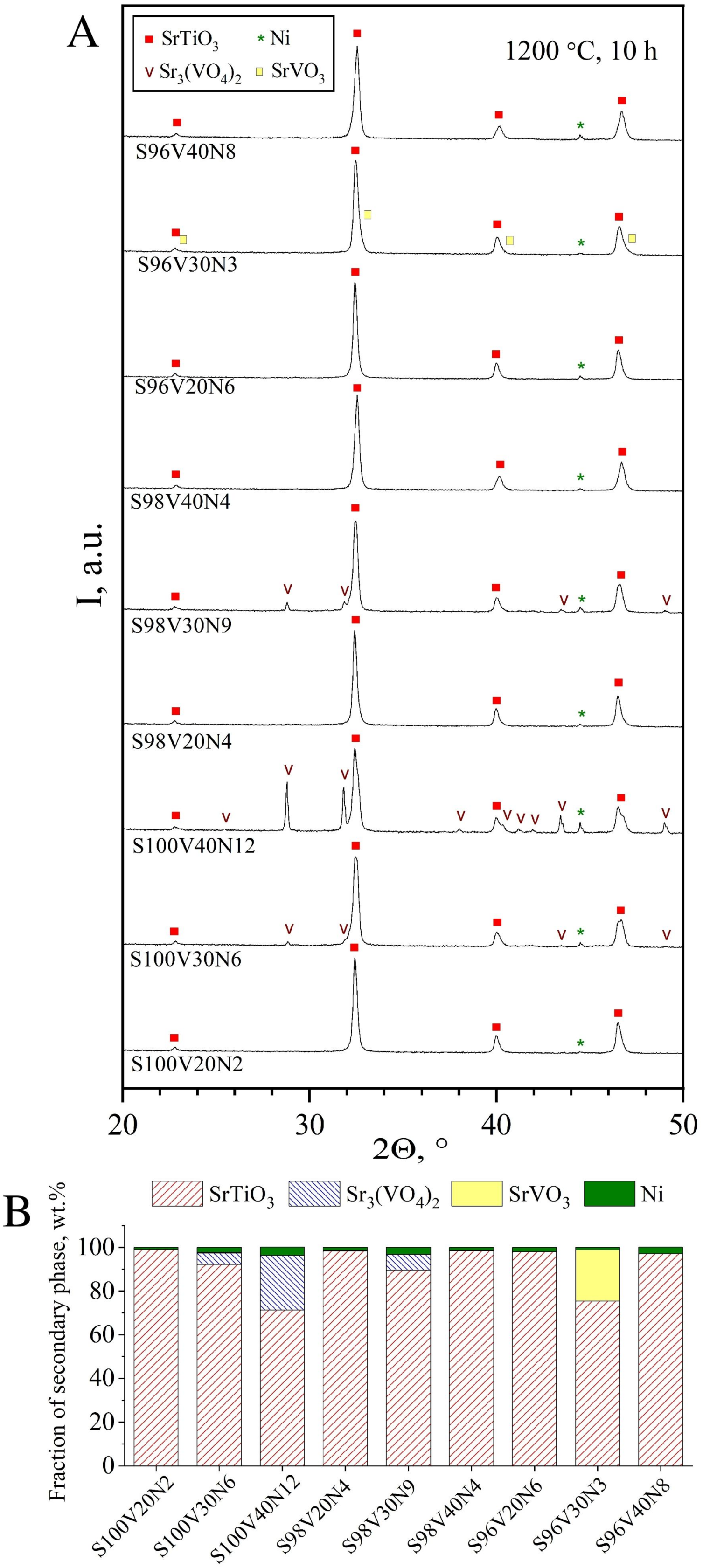

Figure S7. XRD patterns (**A**) and estimated fractions of different phases (**B**) in SVTN samples after firing in a flowing 10%H_2-N_2 atmosphere at 1200 °C for 10 h.

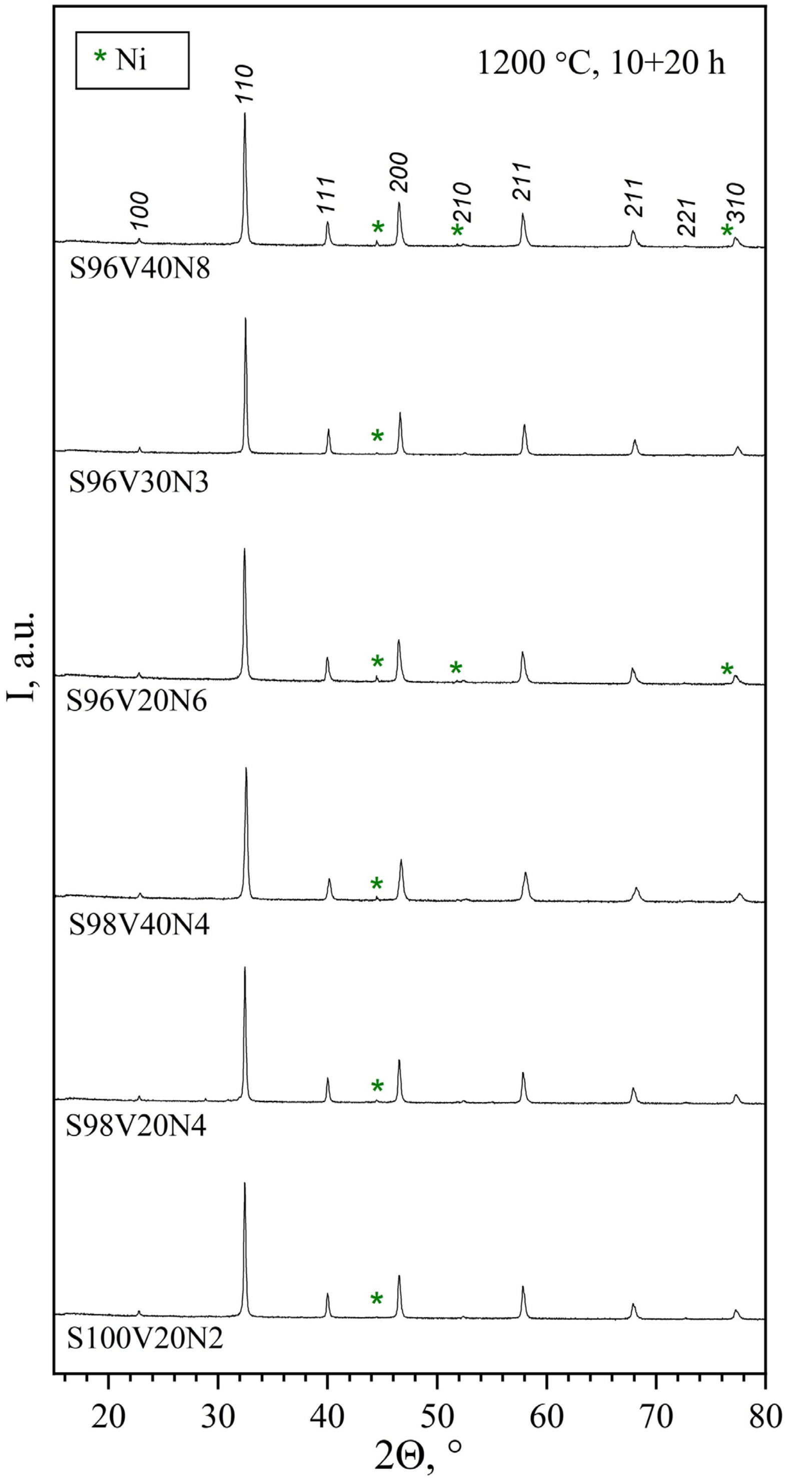

Figure S8. XRD patterns of STVN samples after firing in flowing 10%H_2-N_2 atmosphere at 1200 °C for 30 h.

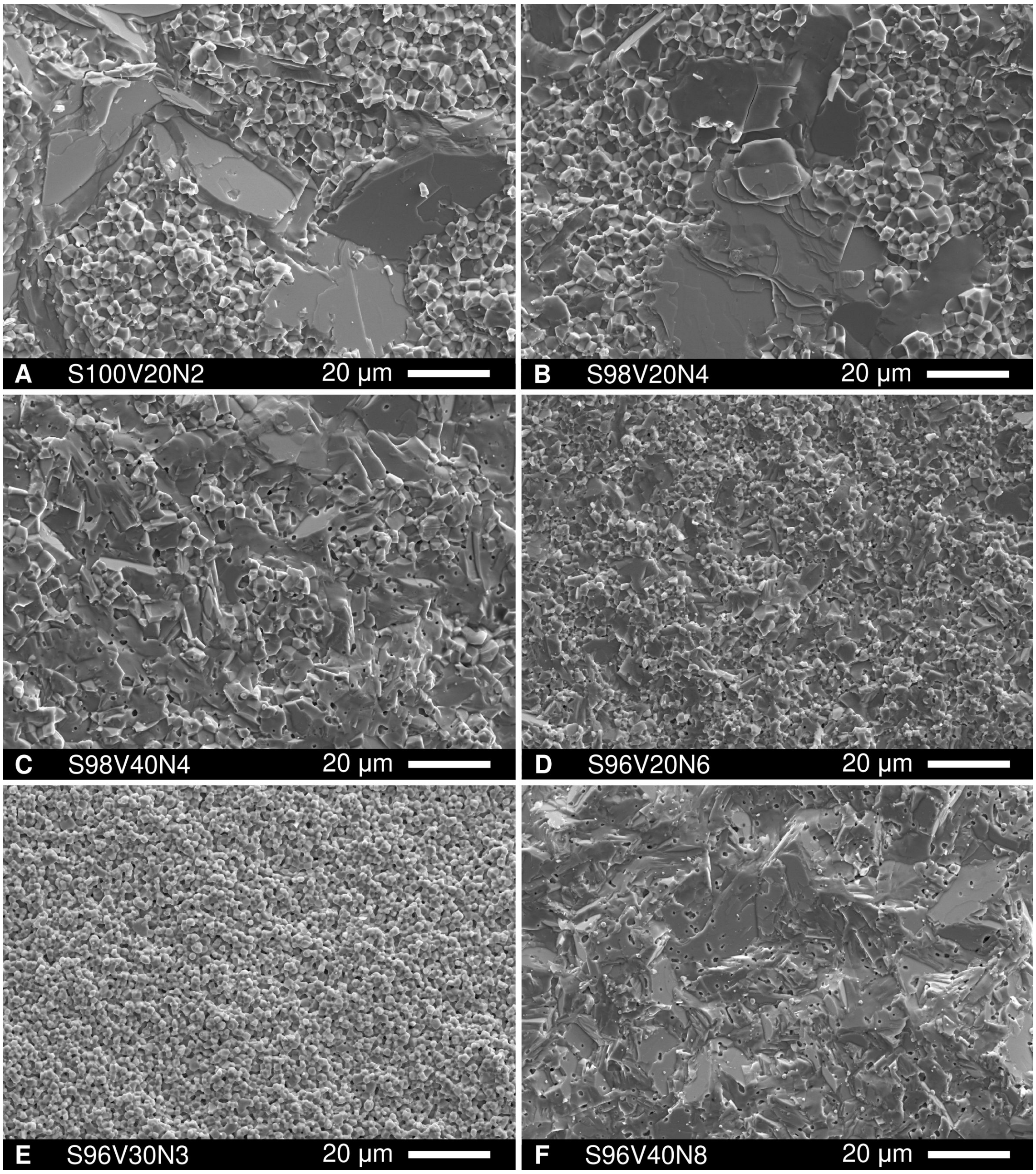

Figure S9. SEM micrographs of fractured cross-sections of STVN ceramics sintered at 1450 °C for 10 h in 10%H_2-N_2 atmosphere.

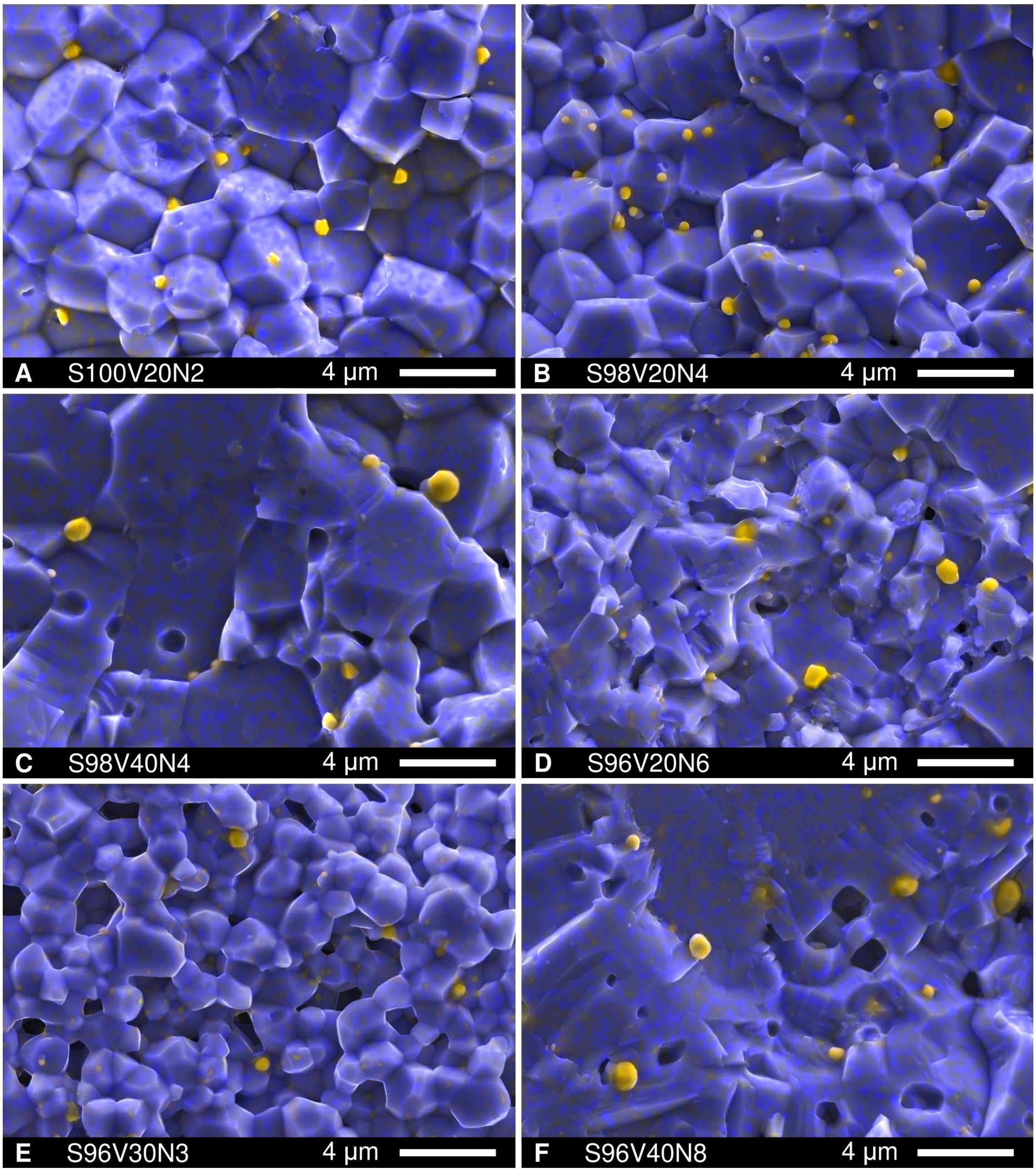

Figure S10. SEM micrographs of the fractured surface of STVN ceramics with overlaid EDS elemental mapping.

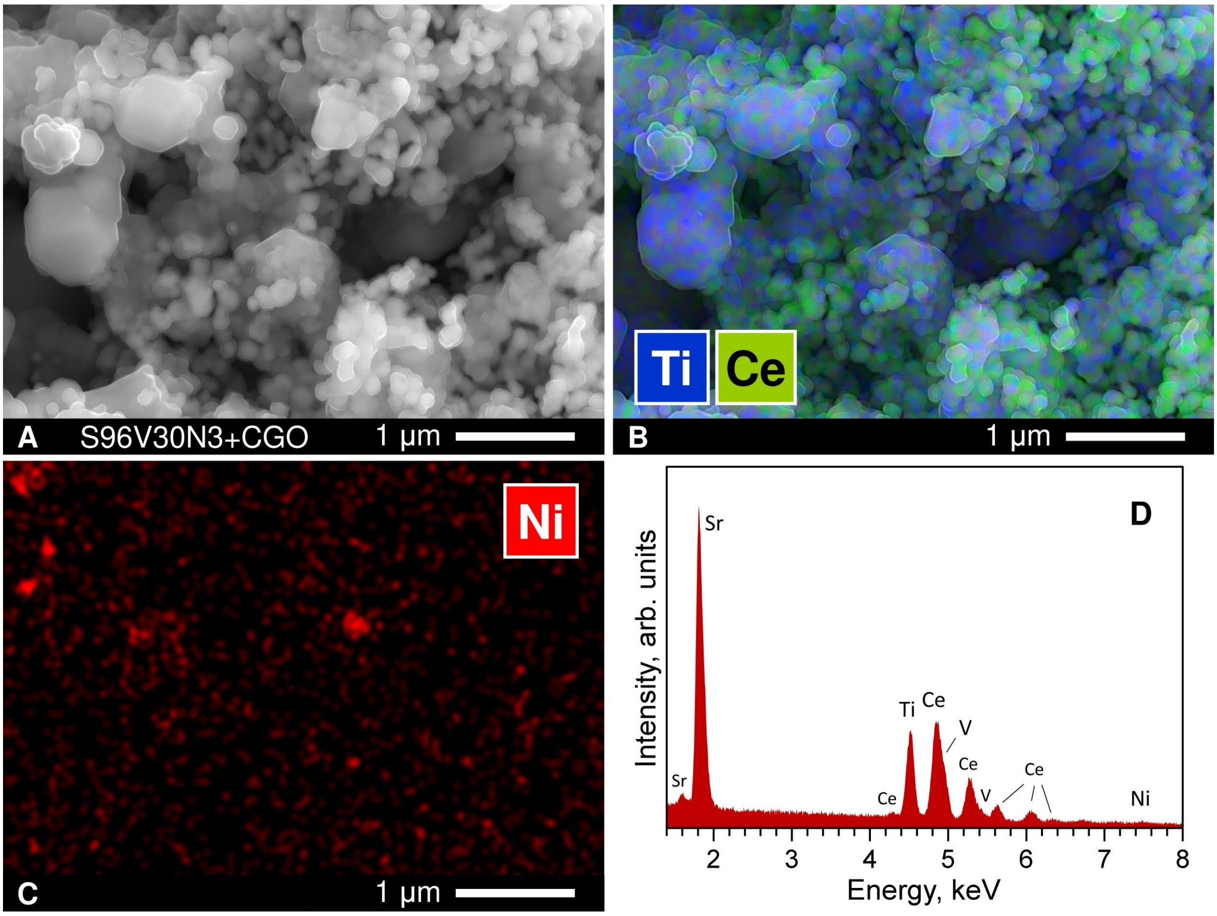

Figure S11. Microstructure of S96V30N3 electrode infiltrated with CGO (26 wt.%): (**A**) SEM image; (**B**) SEM image with overlaid EDS elemental mapping showing the distribution of STVN and CGO phases; (**C**) EDS mapping of Ni distribution; (**D**) corresponding EDS spectrum.

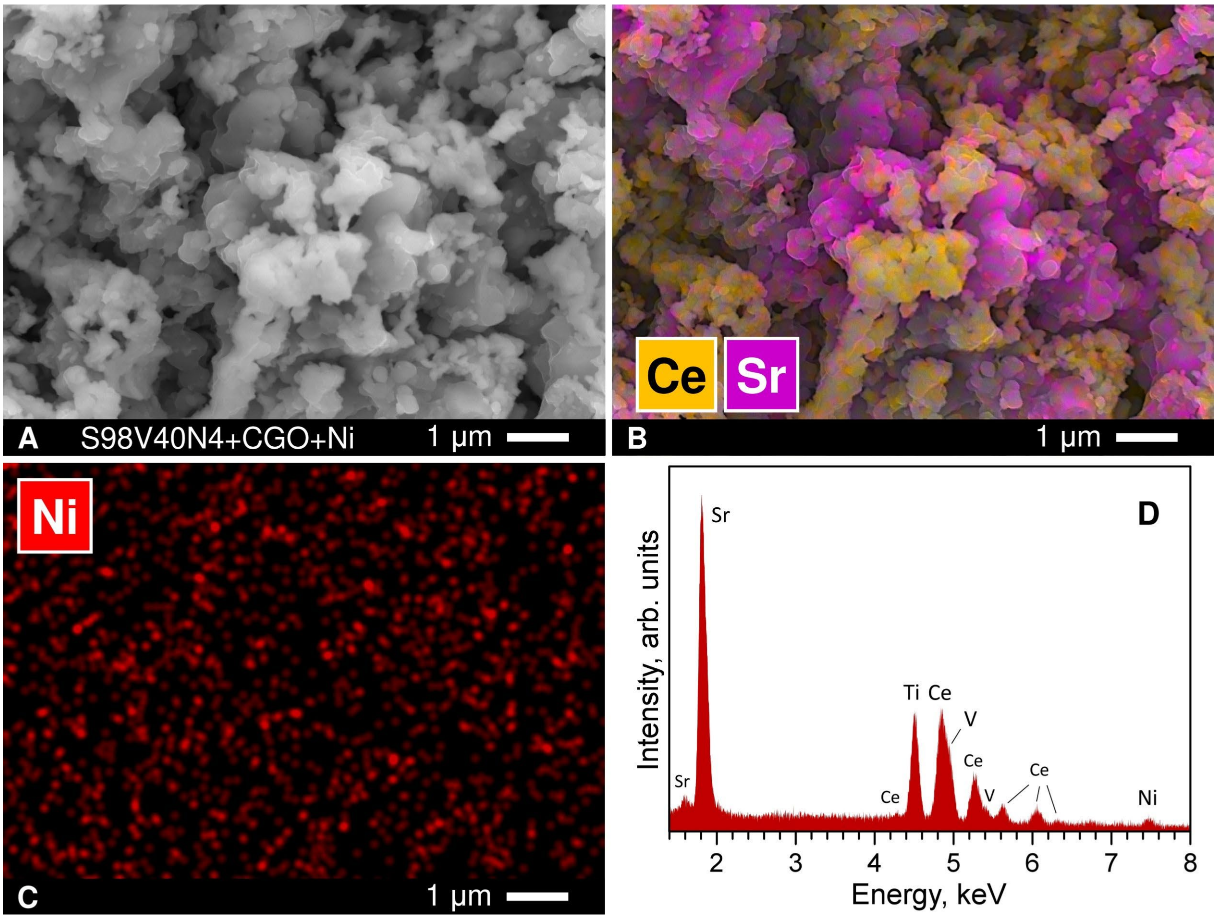

Figure S12. Microstructure of S98V40N4 electrode infiltrated with CGO and Ni (30 wt.%, CGO:Ni = 10:1): (**A**) SEM image; (**B**) SEM image with overlaid EDS elemental mapping showing the distribution of STVN and CGO phases; (**C**) EDS mapping of Ni distribution; (**D**) corresponding EDS spectrum.
